# Connexin 43 mediated collective cell migration is independent of Golgi orientation

**DOI:** 10.1242/bio.060006

**Published:** 2023-10-30

**Authors:** Madhav Sharma, Suvam Mukherjee, Archana Kumari Shaw, Anushka Mondal, Amrutamaya Behera, Jibitesh Das, Abhishek Bose, Bidisha Sinha, Jayasri Das Sarma

**Affiliations:** Department of Biological Sciences, Indian Institute of Science Education and Research Kolkata, Mohanpur, Nadia 741246, India

**Keywords:** Collective cell migration, Connexin 43, Golgi orientation, HeLa 43, HeLa WT, Secretory trafficking

## Abstract

Cell migration is vital for multiple physiological functions and is involved in the metastatic dissemination of tumour cells in various cancers. For effective directional migration, cells often reorient their Golgi apparatus and, therefore, the secretory traffic towards the leading edge. However, not much is understood about the regulation of Golgi's reorientation. Herein, we address the role of gap junction protein Connexin 43 (Cx43), which connects cells, allowing the direct exchange of molecules. We utilized HeLa WT cells lacking Cx43 and HeLa 43 cells, stably expressing Cx43, and found that functional Cx43 channels affected Golgi morphology and reduced the reorientation of Golgi during cell migration. Although the migration velocity of the front was reduced in HeLa 43, the front displayed enhanced coherence in movement, implying an augmented collective nature of migration. On BFA treatment, Golgi was dispersed and the high heterogeneity in inter-regional front velocity of HeLa WT cells was reduced to resemble the HeLa 43. HeLa 43 had higher vimentin expression and stronger basal F-actin. Furthermore, non-invasive measurement of basal membrane height fluctuations revealed a lower membrane tension. We, therefore, propose that reorientation of Golgi is not the major determinant of migration in the presence of Cx43, which induces collective-like coherent migration in cells.

## Teaser

**Summary:** During cell migration, the presence of Connexin 43 reduces the need of Golgi's reorientation to attain directionality.

## INTRODUCTION

Cell migration is a fundamental process that has a pivotal role in early life when embryogenesis occurs (Reig et al., 2014). It is also essential for many physiological functions, e.g. immune surveillance, angiogenesis, and wound healing (Rørth, 2009). Moreover, cell migration plays an essential role in pathophysiological processes such as tumour growth, metastasis and vascular remodelling in various diseases (Friedl and Gilmour, 2009). Directional migration is achieved by establishing front–back polarity in migrating cells (Lebreton and Casanova, 2014). In addition to the asymmetric organization of the cytoskeleton, which is achieved through recruitment and the local synthesis of proteins, polarized cells reorient their secretory traffic towards the direction of migration (Piroli et al., 2019; Zeng et al., 2017). Such exocytic cargos, which originate from the Golgi, contain additional membrane, cell-surface receptors, and extracellular matrix components that are required to maintain the leading edge (Palazzo et al., 2001; Yadav and Linstedt, 2011). Several proteins are involved in controlling cell migration or its modulation (Devreotes and Horwitz, 2015). Among them, connexins, which are classically considered to act as gap junction channel-forming proteins, are known to play an essential role in determining directional migration (Francis et al., 2011). Gap junctions are specialized cell junctions that contain hydrophilic membrane channels that allow the passive diffusion of ions and small molecules between cells (Goodenough and Paul, 2009).

All connexins exhibit a conserved protein structure comprising four transmembrane domains and a cytoplasmic tail localized carboxy terminus with critical regulatory functions (Gourdie et al., 2006; Solan and Lampe, 2005). Connexin 43 (Cx43) is an essential member of the connexin family, ubiquitously expressed across several tissues in a cell-specific manner (Epifantseva and Shaw, 2018; Zhang and Cui, 2017). Its function, however, is not limited to the transport of small molecules. Cx43 plays a significant role in cell migration and polarity by regulating the microtubule network (Francis et al., 2011). Cx43 is synthesized in the ER as an unglycosylated four membrane-spanning monomeric transmembrane protein. Cx43 subunits are post-translationally assembled as hexamers at the trans-Golgi network as Cx43 hexamers are transported in vesicles along the microtubular conduit to reach the cell surface, where they form hemichannels (connexon) and subsequently associate with another connexon to form GJ channels (Epifantseva and Shaw, 2018). Studies have shown that polarized Golgi is vital for normal developmental processes and directed secretion of extracellular factors, responsible for effective cell migration. In a polarized cell, Golgi is pericentrosomally oriented towards the direction of migration (Yadav et al., 2009). Intracellular remodelling of Golgi happens through the microtubule, with the latter tethering Golgi at the polarization region (Mimori-Kiyosue et al., 2005).

Leader cells are the first to explore the tissue microenvironment during cell migration (Etienne-Manneville and Hall, 2001). Thus, they contribute to the navigation of follower cells. For the leader cells, directing the Golgi towards any direction of the mechanical void makes the Golgi directed towards the scratch in a scratch assay (Bindschadler and McGrath, 2007). By secretion of extracellular matrix (ECM), leader cells create a substratum for the followers and make it easy for them to migrate (Vitorino and Meyer, 2008). Follower cells also influence the leader cells through the cell surface molecules like cadherins (Barriga et al., 2013; Becker et al., 2013; Theveneau et al., 2010), ephrins (Astin et al., 2010; Batson et al., 2014; Villar-Cerviño et al., 2013), members of the polar cell planarity complex, i.e. Frizzled (Carmona-Fontaine et al., 2008), WNT (Mayor and Theveneau, 2014), PTK7 (Shnitsar and Borchers, 2008) and syndecan 4 (Matthews et al., 2008), thereby encouraging directed migration of leader cells. Thus, in collective cell migration, leaders and followers influence each other through their continuous, directed motion (Arima et al., 2011; Jakobsson et al., 2010). In systems like keratocyte cell sheets, a lateral connection between neighbouring cells and actin cables across multi-cellular length scales coordinate the coherent movement of the front (Chakraborty et al., 2022).

In contrast, in single cells, the coherence of the lamellipodia extension is responsible for converting random migration to directed cell migration (Keren et al., 2008; Ofer et al., 2011). A front–back gradient of membrane tension has been reported and is believed to be critical to channel forces in one direction (Lieber et al., 2015). Directing secretory machinery (of the Golgi apparatus) towards the leading edge assures timely membrane delivery through exocytosis and maintenance of the front tension, which would otherwise keep increasing due to actin polymerization forces (Gauthier et al., 2011). Despite this role of the Golgi apparatus, multiple studies report Golgi to be not necessarily oriented towards the leading edge of migrating cells (Natividad et al., 2018). There exists a clear gap in our understanding of the dependency of migration on Golgi reorientation. In particular, enhancing cell–cell communication through GAP junctions can fundamentally alter information flow between cells, reducing mechanical signals’ impact, and increasing chemical connectivity. It remains unexplored if cells depend on front–back tension gradient and Golgi reorientation when cell–cell communication is established.

Thus, although Golgi orientation in migrating cells plays a crucial role, the underlying mechanism and its correlation with the presence of functional GAP junctions need to be adequately evaluated. Towards this, our study using HeLa WT (Cx43-/-) and HeLa 43 (Cx43+/+) demonstrated that expression of Cx43 is associated with morphological alteration of the Golgi apparatus in HeLa 43 cells. Interestingly, we also observed the expression of vimentin, mesenchymal cell protein, and DJ-1 protein, a known antioxidant protein indicating more inclination towards the mesenchymal character, suggesting that in the tussle between the presence of gap-junctions and mesenchymal characteristics, functional gap junctions in HeLa 43 cells slowed down the collective cell migration. The basal actin was imaged using total internal reflection fluorescence (TIRF) microscopy and revealed how Cx43 affects the migrating cell surface. Interference reflection microscopy (IRM) was used to assess the concomitant changes in the mechanics of the basal membrane in the presence of Cx43 that might be linked to the slower and coherent migration.

## RESULTS

### Golgi orientation is random in the absence of migration

**Fig. 1.**
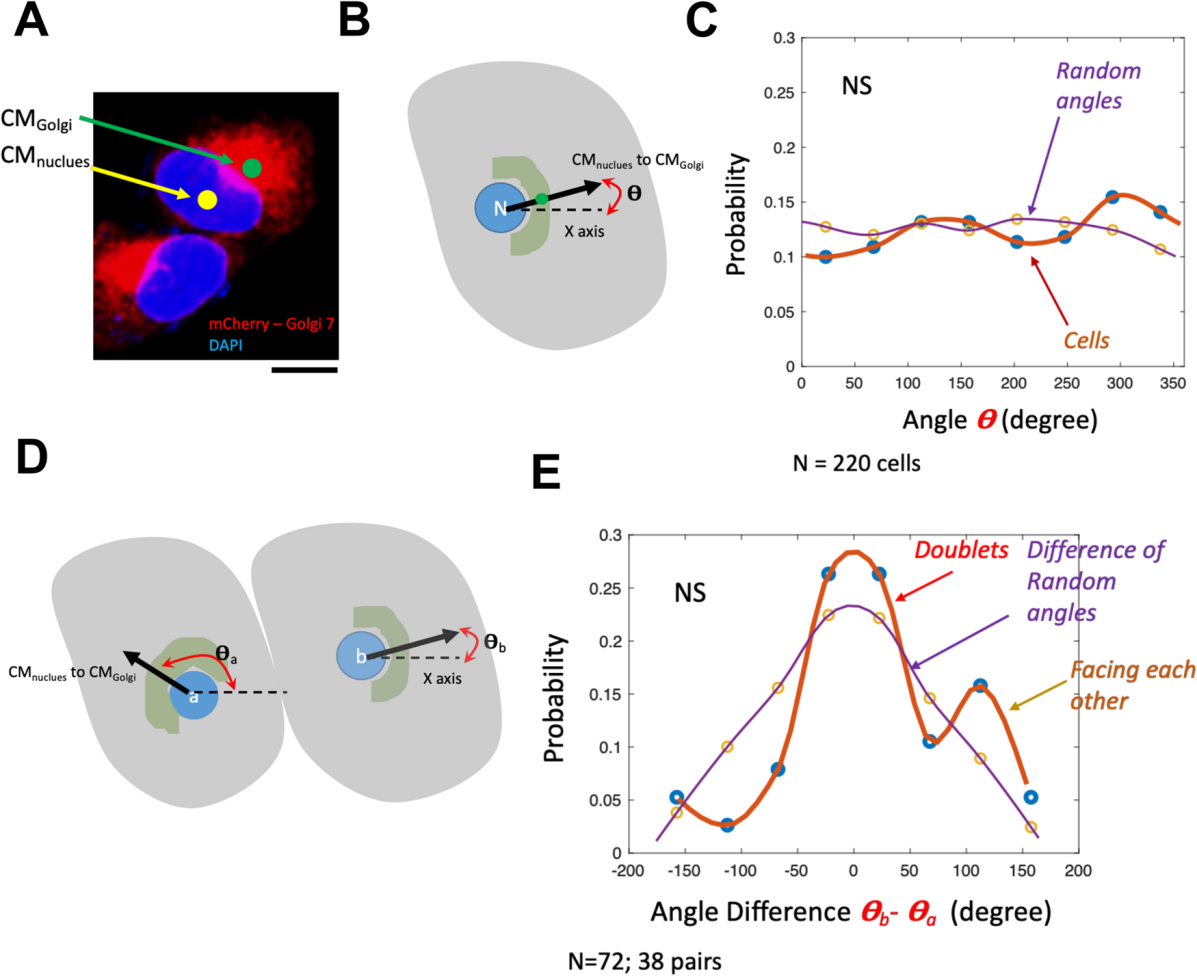
**Quantifying Golgi's orientation.** (A) Representative image of cells expressing mCherry-Golgi 7 (red) and stained with Hoechst 33342 (blue). Scale bar: 10 µm. (B) Schematic of angle measurement from CM_Nucleus_ and CM_Golgi_ measured for nucleus and Golgi using image analysis. (C) Angle distribution and its comparison to normally distributed random numbers in the same range. No significant difference was found, as expected. Wilcoxon rank-sum test was performed to test for significance. *N*=220 cells. (D) Schematic representation of angle measurements for doublets (cell represented in grey, Golgi represented in green, and nucleus represented in blue). For each cell, the angle (***θ***) subtended at the x-axis, by the line joining its CM_Nucleus_ to CM_Golgi_ is first measured. Cell whose CM_Nucleus_ is at a bigger x value (or cell is to the right) is termed as the ‘b’ and the other one as ‘a’. (E) Distribution of ***θ***_***b***_−***θ***_***a***_ and distribution of difference of uniformly distributed random angles in the same range as observed for cells. Angle differences obtained from cells were not significantly different from the difference of random angles as tested by Wilcoxon rank-sum test.

The Golgi apparatus can be distributed in different forms in cells. However, HeLa wild-type (WT) cells usually display a clustered distribution, as visible in the images of HeLa WT cells expressing mCherry-Golgi7 (Fig. 1A) which marks the trans-Golgi (Qasba et al., 2008). To quantify its orientation (Fig. 1B), the centroid (CM_Nucleus_) of the image of the nucleus is joined to the centre of mass (CM_Golgi_) of the Golgi to get the angle. Without any ‘front’, no particular direction can be used as a reference. Hence, the x-axis of the image is taken as the reference. The angle is measured for it (Fig. 1B). Once such angles are obtained for all cells in a particular dish (sharing a common x-axis), distributions do not show any angle favoured. Pooling over multiple dishes from multiple trials (Fig. 1C), the distribution was statistically not significantly different from a uniformly generated random numbers distribution.

To understand if cell–cell interactions were enough to reorient Golgi – even in the absence of cues from the wound – we next analysed images of cell ‘doublets’ (Fig. 1D). The centroid of the binary image of the nucleus (marked as CM_nucleus_) was joined to the centre of mass (CM) of the Golgi (computed from the grayscale image to consider intensity variations) to get the angle with respect to the x-axis of the image. For each cell, this angle (θ) was first measured and then compared between cell pairs in the doublets (θ_b_-θ_a_). The cell whose CM_nucleus_ was at a more considerable x value (or cell is to the right) was called ‘b’ and the other one is ‘a’. The distribution of θ_b_-θ_a_ and the distribution of the difference between two uniformly distributed random angles in the same range were compared and statistically tested. Angle differences obtained from cells were not significantly different from those of random angles (Fig. 1E) as tested by the Wilcoxon rank sum test in MATLAB.

Thus, for doublets, we found that there was a slightly higher probability of finding cells facing each other, than randomly; however, the data remained statistically non-significant from simulated data mimicking the random direction of the cells. This established that measured orientations were not substantially influenced by how neighbouring cells are arranged without any spatial cue, like in a wound.

### Stacked Golgi apparatus in HeLa sensitive to Cx43

**Fig. 2.**
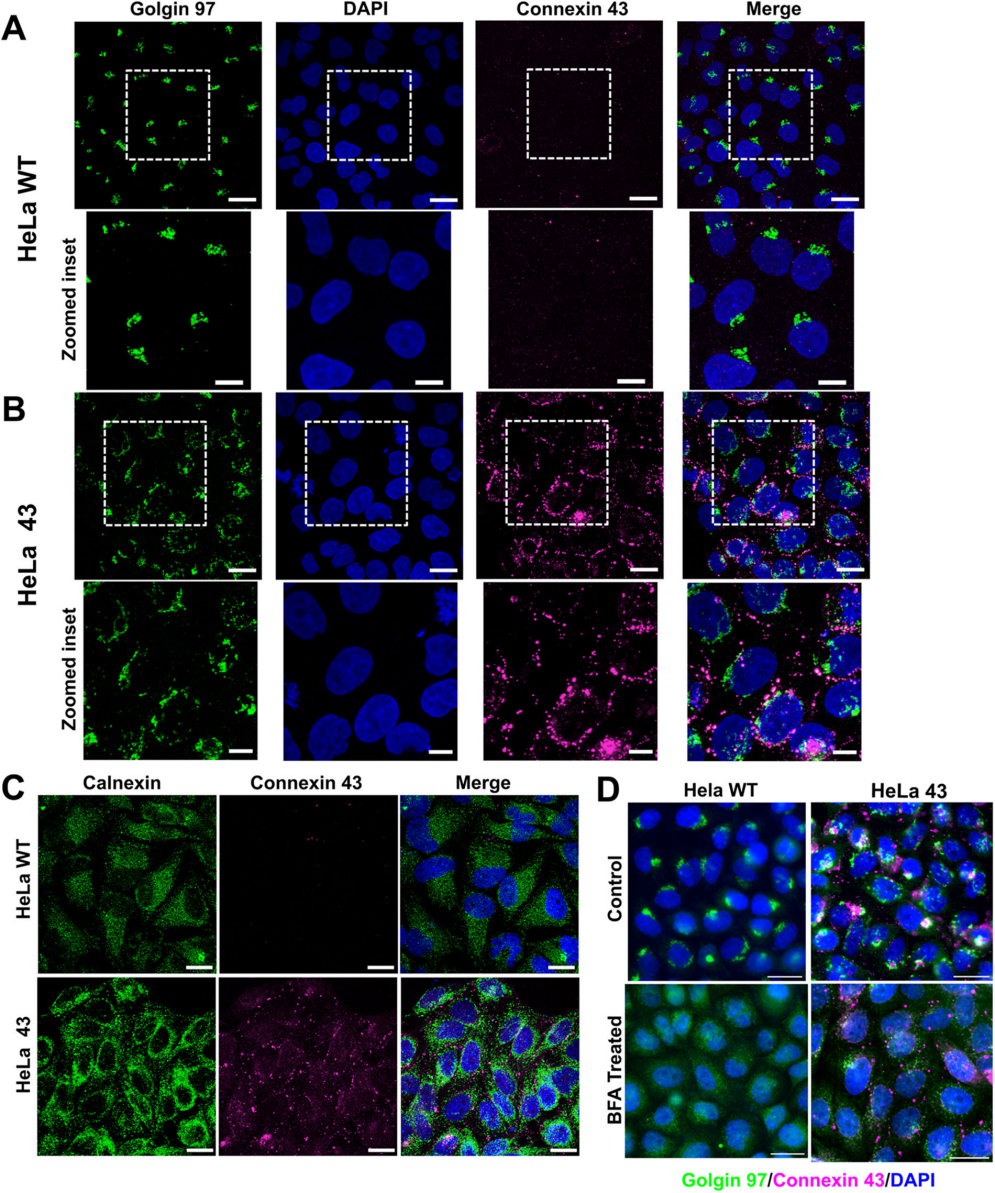
**Characterizing HeLa WT and HeLa 43.** (A) Confocal images of HeLa WT. (B) Confocal images of HeLa 43 highlighting Golgi and Cx43 distribution. Zoomed insets provide better resolution of the altered morphology of Golgi in HeLa 43 in A and B. Scale bars: 50 µm; zoomed-in images/inset=20 µm. (C) IF with calnexin antibody reveals no particular difference in ER in the two cell lines. (D) BFA alters Golgi distribution in both cell types. C,D, scale bars: 30 µm.

Golgi apparatus is the protein glycosylation and transport machinery of the cells and plays an important role in membrane trafficking. HeLa WT cells, which are epithelial carcinoma cells, and lack Cx43, were used to understand its role in cell migration by comparing with a stable line of HeLa WT expressing Cx43 (Das Sarma et al., 2002), and termed HeLa 43. These cells lacked other connexins like Connexin 32 and Connexin 36 (Fig. S1). Golgi apparatus of HeLa WT cells were characterized using Golgin 97 protein, predominantly present in trans-Golgi network (TGN) and plays critical roles as a tethering molecule associated with tubulovesicular carriers during vesicle trafficking and in maintaining the Golgi integrity (Hsu et al., 2018). Immunofluorescence with anti-Golgin 97 antibodies indicates condensed Golgi in HeLa WT (Fig. 2A). Immunofluorescence also confirmed that HeLa WT was negative for Cx43 expression. To understand the role of Cx43 in cell migration, HeLa 43, HeLa WT cells stably expressing Cx43 were used for comparison. Immunofluorescence data suggested that cell surface expression of Cx43 was associated with diffused staining of Golgin 97, denoting altered Golgi structure. The Golgi appeared less condensed in HeLa 43 as opposed to HeLa WT (Fig. 2B), suggesting that the exogenous expression of Cx43 at the cell surface, as well as intracellular retention, may cause the altered distribution of Golgi stacks. Also, HeLa 43 cells displayed gap junction of Cx43 in the form of puncta as shown (Fig. 2B). Endoplasmic reticulum (ER) of HeLa WT and HeLa 43 were also characterized using anti-Calnexin antibody (a chaperone exclusively present in the ER), and indicated ER is more conical shaped (Fig. 2C). Interestingly, the cis-Golgi (Fig. S2) did not show a less condensed structure in HeLa 43 as it did in trans-Golgi.

To validate the influence of Golgi's orientation with or without Cx43 on migration, we used Brefeldin A (BFA) to create ER stress and thereby convert a stacked Golgi to the distributed vesicular organization in the cell (Fig. 2D).

**Fig. 3.**
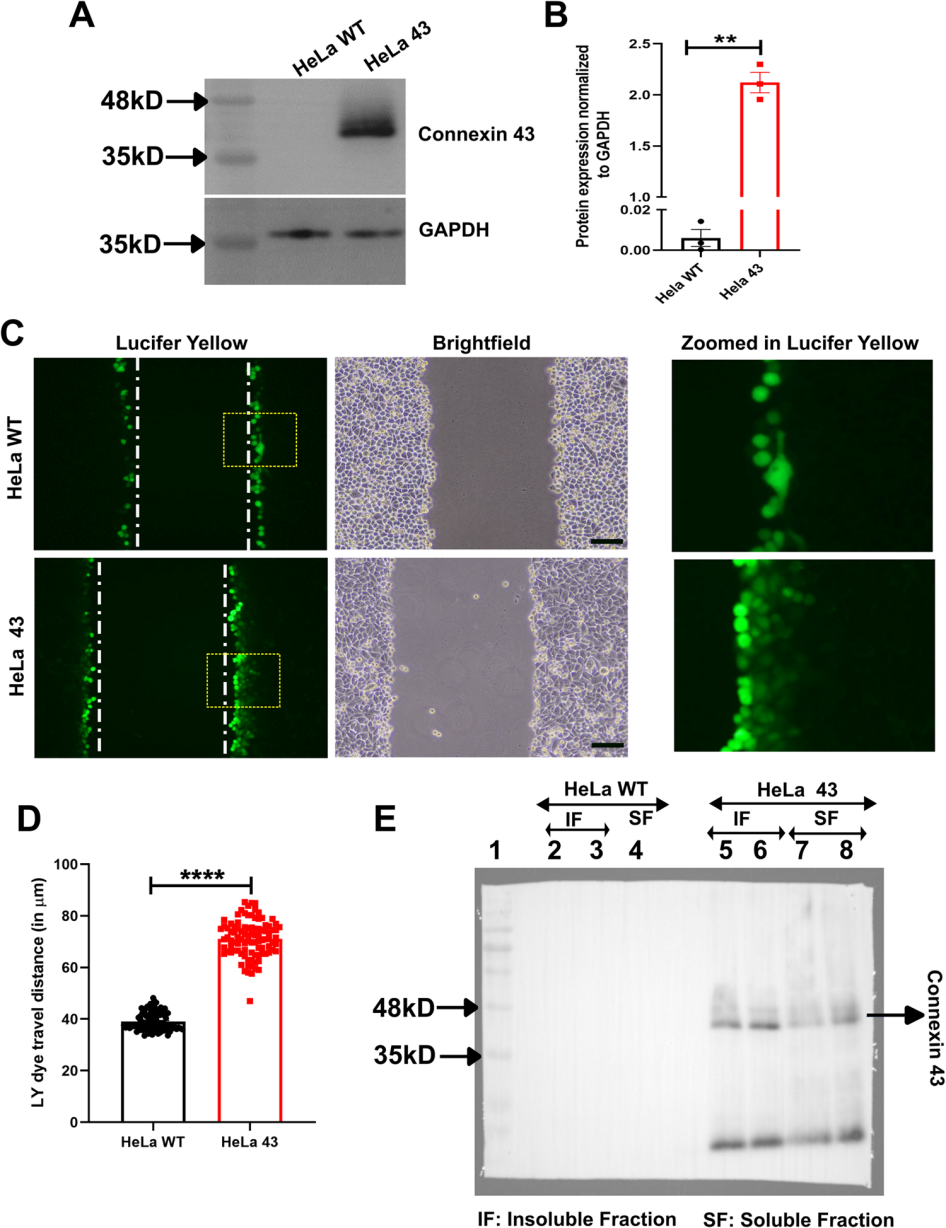
**Evaluating presence and functionality of gap junction.** (A) Western blots showing a higher abundance of Cx43 in HeLa 43. (B) Quantification of western blot. (C) Scrape loading assay depicted with representative scrapes with LY shown in green. Vertical white lines denote the scrape line. Rectangular ROIs outlined regions expanded in the rightmost column. Scale bars: 100 μm. (D) Quantification of LY dye transfer, showing dye transfer in HeLa 43 is significantly higher than in HeLa WT, thereby depicting the presence of functional gap junctions in HeLa 43. (E) Comparison of Tx-solubilisation between HeLa WT and HeLa 43. Lane 2 and 3, HeLa WT insoluble fraction; 4 HeLa WT soluble fraction; 5 and 6, HeLa 43 insoluble fraction, and 7 and 8, HeLa 43 soluble fraction. For statistical significance (B,D), asterix (*) represents differences that are statistically significant by Student's unpaired *t*-test analysis (**P<0.01, ****P<0.0001). The error bars represent s.e.m.

Immunoblot assay (Fig. 3A,B) using anti-Cx43 antibody confirms the presence of Cx43. In contrast, HeLa WT was found to be negative for Cx43 expression both in immunofluorescence and immunoblot assays confirmed by quantification (Fig. 3B).

### HeLa 43 forms functional gap junctions

To check the functional gap junction, the scrape loading/dye transfer technique was used, which is a simple, functional assay for the simultaneous assessment of gap junctional intercellular communication (GJIC) between adjacent cells in a large population of cells. As Cx43 channels are permeable to Lucifer Yellow (LY) (<1 KD), a small molecular dye that moves from cells to the neighbouring cells via gap junctions, the green colour dye transfer in the HeLa 43 cells indicates the presence of functional gap junctions formed by Cx43 as opposed to HeLa WT, which does not establish functional gap junctions (Fig. 3C,D). Furthermore, Cx43 assembled into gap junction plaques is resistant to 1% Triton X-100 solubilization at 4°C, while remaining Cx43 dissolves in the Triton X-100 soluble fraction. Previous reports have demonstrated that the mutant non-functional form of Cx43 is not detected in the insoluble fraction indicating its inability to form functional gap junctions. However (if expressed) it retains in the intracellular compartments and can be detected in Triton X soluble fraction (Das Sarma et al., 2002). Therefore, Triton X-100 solubilization was performed to determine the retention of Cx43 in the intracellular compartment instead of it being trafficked to the cell surface to form gap junction plaques. The membrane fraction was isolated from cells and was solubilized using 1% Triton X-100 at 4°C, separated into detergent-soluble and -insoluble fractions, and probed for Cx43. HeLa 43 showed that most Cx43 was pooled more in the Triton X-100 insoluble fraction rather than the soluble fraction. Hence, the insoluble/soluble fraction ratio of Cx43 expression was significantly higher in HeLa 43 (Fig. 3E). This experiment demonstrated that a small amount of Cx43 retains in the intracellular compartment (triton soluble fraction). In contrast, a large fraction of Cx43 was observed in the insoluble fraction, which denotes that Cx43 forms insoluble gap junction plaque at the cell surface.

### Golgi reorientation during migration is lower in HeLa 43

**Fig. 4.**
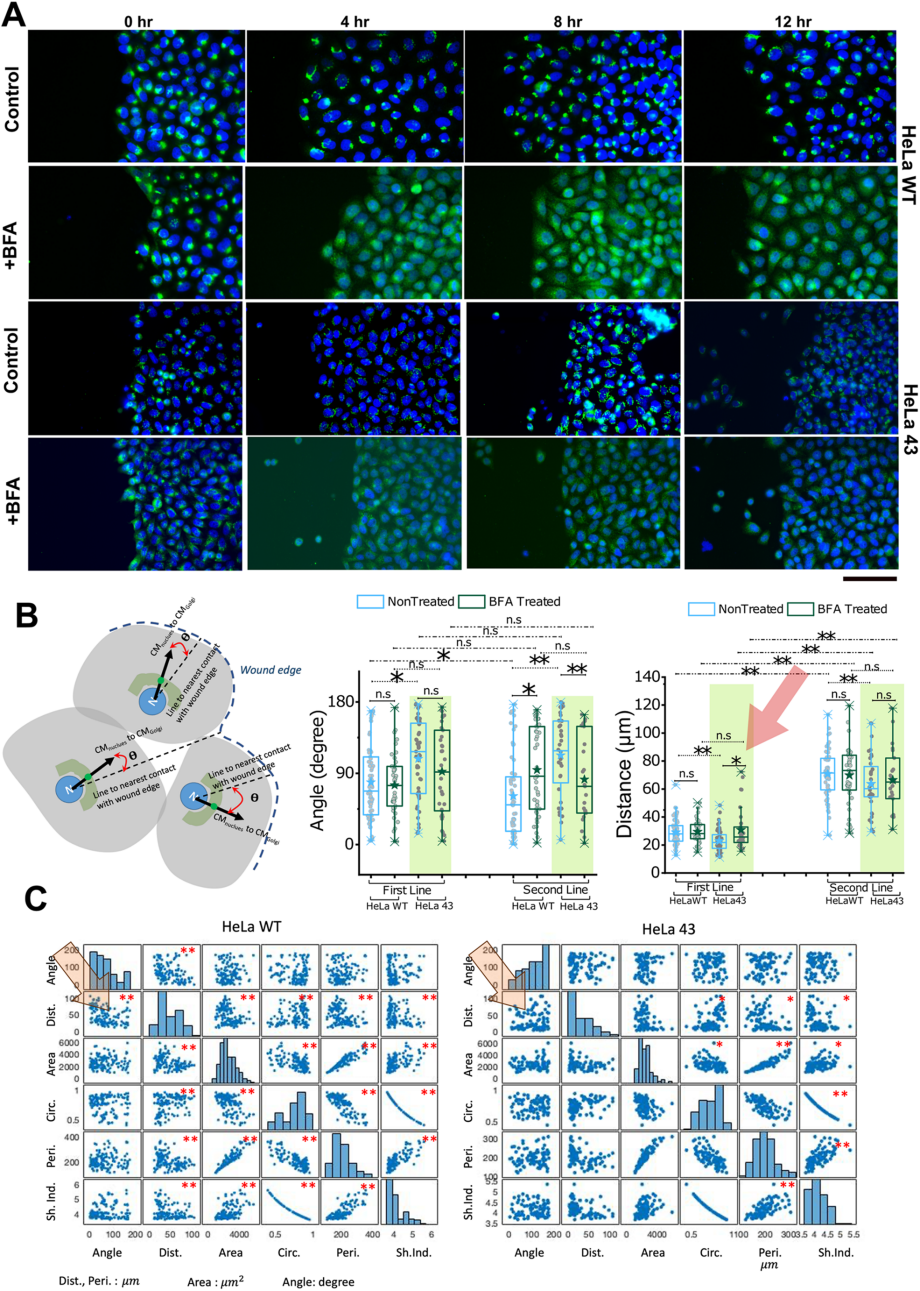
**Scratch assay and quantification.** (A) Depiction of directional movement of Golgi [using anti-Golgin 97 antibody (green) and Hoechst 33342 stain for DNA (blue)] in HeLa WT and HeLa 43 by immunofluorescence. Scale bar: 150 μm. (B) Left: Schematic depicts the method used to measure the angle between the cell's nucleus-Golgi axis and shortest line connecting it to the wound edge. Centre: Comparison of Golgi orientation after 12 h of migration. Right: Comparison of reorientation in leading cells (first line) and following cells (line 2). For statistical significance, the Mann–Whitney *U*-test was performed, *P<0.05, **P<0.001. Representative of two independent experiments. (C) Correlation between different parameters [angle, distance (Dist), area, circularity (Circ), perimeter (peri) and shape index (Shp. Ind.)]. For statistical significance, the Wilcoxon rank-sum test (equivalent to Mann–Whitney test) was performed, *P<0.05, **P<0.001. Representative of two independent experiments. Number of cells used: HeLa WT(Control): 127 cells; HeLa WT(BFA): 85 cells; HeLa 43(Control): 87; HeLa 43(BFA): 57 cells.

We next set up the scratch assay in which an asymmetry of neighbours would be introduced. To quantify the reorientation of Golgi as the migration progresses, using immunofluorescence on samples fixed at different time points (Fig. 4A), we imaged the Golgi's distribution in cells that face the wound (termed as ‘first line’ cells) as well as cells not directly exposed to the scratch but separated by a line of cells (termed ‘second line’ of cells).

To quantify Golgi reorientation with respect to the wound, the angle between the cell's nucleus-Golgi axis and the shortest line connecting it to the wound edge was found using MATLAB as explained schematically (Fig. 4B). The angles were measured from −180 to 180 and the absolute values were used. If the Golgi apparatus was oriented towards the wound, the absolute angle would be close to 0; if it faced the opposite direction, it would be 180.

Such images were later analysed to yield multiple parameters that may link Golgi's reorientation to cellular morphometric data, like cell spread area, perimeter, shape index, distance from wound and Golgi's orientation with the wound. However, to decipher the role of Cx43, we then demonstrated similar experiments in the system with enhanced Cx43 – the HeLa 43 cells.

To understand the role of Golgi orientation and its correlation with Cx43 on cell migration, BFA, which is an inhibitor of intracellular protein transport and leads to the blockade of protein transport to the Golgi complex (GC) and accumulation of proteins in the ER, was used.

Comparing orientation in HeLa WT cells from the first two lines with that of HeLa 43 cells (Fig. 4B, centre), we found that HeLa WT cells were more oriented towards the wound than the HeLa 43 cells after 12 h of cell migration in the scratch assay. However, it must be noted that both cells were weakly reoriented and hence a late time-point of 12 h was chosen for the comparison of the angles. Weaker reorientation of HeLa 43 cells is in line with their lesser compact distribution. On BFA treatment, the level of reorientation of the first line of cells did not show any significant change either in HeLa WT or HeLa 43 cells. However, with both first line and second line cells, while untreated cells of HeLa 43 were less oriented than HeLa WT, when treated with BFA, HeLa WT and HeLa 43 were similar. Hence the presence of Cx43 did not affect the reorientation of Golgi in the presence of BFA.

We next checked if the first-line cells responded differently than the following cells. We observed that the second-line cells for HeLa WT were more aligned (towards the wound) than the first-line cells. However, such a trend was not seen in HeLa 43 cells.

Interestingly, HeLa 43 remained less oriented than HeLa WT when their first or second lines were separately compared. It was also interesting to note that BFA affected the orientation of the second line of cells. It further randomized the HeLa WT while giving more orientation to HeLa 43. BFA-treated HeLa 43, however, was not statistically different from BFA-treated HeLa WT in their orientation angle in the second line cells as seen for the first line cells.

This data first confirms the less reorientation shown by HeLa 43 in both for the first and second line of cells. Note that a similar difference between first and second-line cells was not observed in HeLa 43, showing that cells were more similar despite being in the first versus second line. These data revealed that reorientation was higher in second-line cells for HeLa WT, emphasizing that the cause was not directly linked to the availability of free space evidenced by first-line cells. However, since the first-line cells could ‘face’ the wound at a much wider angle, we could not yet conclude that there was any distance dependence in the reorientation. Therefore, we next compared the other parameters measured before evaluating the correlations between them.

Among the various other single-cell parameters (Fig. S3), the shape index (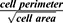) has been used in the literature to capture the state of jamming of cells (Atia et al., 2021). The state of jamming indicates how trapped or stuck cells are while in the collective system (here, monolayer) and in this study could compare the effect of altered levels of Cx43 and Golgi orientation on one aspect of motility. Several studies have observed a common factor – the shape index faithfully reflects such a dynamic property of cells. It has been observed that lower the shape index, the more jammed the cells (Park et al., 2015). We found that HeLa 43 were not different from HeLa WT in its state of jamming, and neither did BFA affect the state of jamming in either HeLa WT or HeLa 43. All cells were primarily unjammed. However, we found that second-line cells were more jammed than the first-line cells, which was also expected since the first line had free space at their front.

**Fig. 5.**
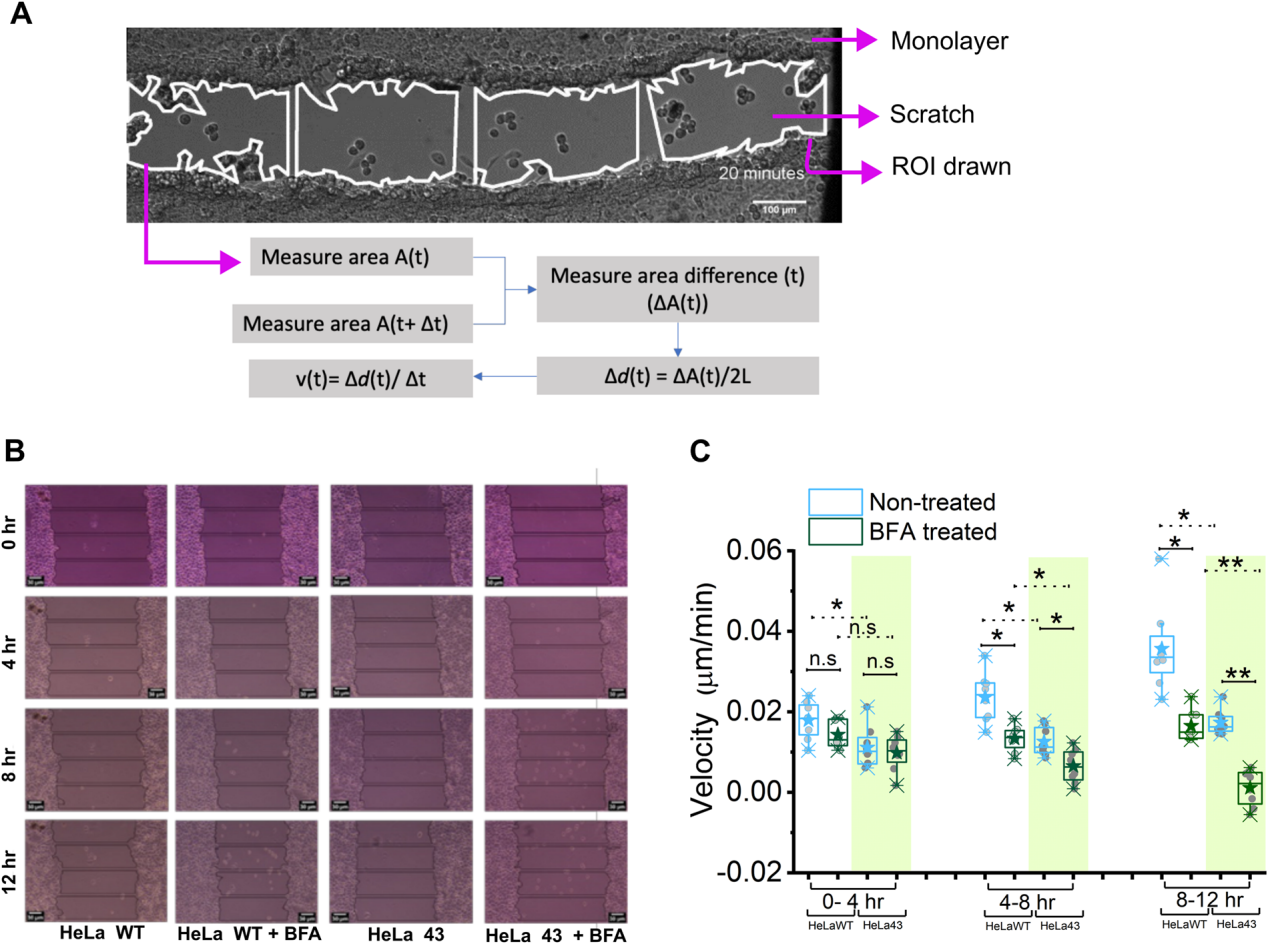
**Effective velocity of cells during scratch assay**. (A) Representative image and strategy used to measure velocity. Scale bar: 100 μm. (B) Representative image and sectioning of the image. Scale bar: 30 μm. (C) Comparison of velocity measured in different conditions as described. Representative of two independent experiments. *N*=8 regions (of scratch assays). For statistical significance (C), the Mann–Whitney *U*-test was performed; ns, *P*>0.05; **P<0.001.

Evaluating distance of centroid of the nucleus of each cell from wound-edge showed that first-line HeLa 43 cells were marginally, but significantly, closer to the wound than HeLa WT (Fig. 5B, right), and this was not random because on treatment with BFA, the placement is lost, and BFA-treated HeLa WT and HeLa 43 cells were similar in their distances. This points to the fact that while leading HeLa WT cells have more oriented Golgi, leading HeLa 43's Golgi is less oriented but nearer the plasma membrane facing the wound. For second-line cells, this variation was not present anymore.

Comparison of circularity (Fig. S3) (1 for circle, lower for non-circular shapes) revealed that while second-line cells were more circular than first-line cells under all conditions, there was a significant change between HeLa WT and HeLa 43 cells. This was expected since first-line cells had more space for lamellipodia of various shapes that alters circularity. This also resulted in less perimeter and area in the second-line cells.

To properly quantify the correlation between parameters, we find the correlation coefficient in MATLAB and statistically test the significance of the correlation (Fig. 5C; Table ST1, ST2). As expected, area, circularity, perimeter, and shape index are correlated since shape index is derived from perimeter and area, and cell size would affect both perimeter and area resulting in correlation.

However, interestingly, the distance of the cell from the wound was negatively correlated with the angle of orientation in HeLa WT. Since this is also seen in first-line cells, this reconfirms the hypothesis that alignment was more required when the wound edge and cell centre were farther away. The correlation was lost in HeLa 43 cells, where lower orientation, we believe, was compensated by lowering distance from the edge.

### Front motility of HeLa 43 is slower but more coherent than HeLa WT

To check the functional implication, we next quantified the velocity of the moving front of cells at different time points after wounding using the scratch assay. The analysis method adopted (Fig. 5A) involved dividing the frame into four equal-length regions of interest (ROIs). The ROIs were drawn following the cell edges such that their area represented the area of the section of wound. With time, as the cell edges would move closer, redrawn ROIs would capture the reduction in area. Since the area could be measured most robustly, area change was used to derive the effective average velocity from ROIs, as shown in Fig. 5A. Multiple ROIs and scratches over different trials were used for comparisons.

We found that (Fig. 5B,C; Table ST3) HeLa 43 was always slower than HeLa WT cells. Although at early time points BFA did not hamper mobility, at intermediate and later (4-8 and 8-12 h) time points, BFA slowed down HeLa WT as well as HeLa 43. However, the difference of median velocity of untreated and BFA-treated cells was higher for HeLa WT than HeLa 43 for all time intervals [(0-4 h: 0.0053 (HeLa WT) versus 0.00013 (HeLa 43) µm/min; 4-8 h: 0.011 (HeLa WT) versus 0.005 (HeLa 43) µm/min; 8-12 h: 0.019 (HeLa WT) versus 0.015 (HeLa 43) µm/min)]. At the 8-12 h time interval BFA's effect was found to be most effective, therefore, justifying the use of 12 h time point for evaluating the effect on orientation angle (Fig. 4B, centre). Thus, although motility of both cell types was finally slowed by BFA, both the higher orientation of the Golgi towards the wound, and the median velocity for HeLa WT was more sensitive to BFA-treatment than HeLa 43.

However, interestingly, for HeLa 43 cells, variations in velocity [quantified as standard deviation (SD) in measured velocity] between regions was comparable at the early time interval of 0-4 h (both 0.005 µm/min) but was higher in HeLa WT than HeLa 43 for both 4-8 h (HeLa WT: 0.006 versus HeLa 43: 0.004 µm/min) and 8- 12 h (HeLa WT: 0.011 versus HeLa 43: 0.003 µm/min) time intervals. As evident, even with progression of migration, SD of velocity increased twice for HeLa WT cells but decreased for HeLa 43. This showed that although HeLa WT was faster in moving, its migration was less spatially coherent and therefore had more inter-regional velocity variations along the wounds. These variations did not increase as much in BFA-treated HeLa WT cells (0.003–0.004 µm/min) as in untreated HeLa WT cells (0.005–0.011 µm/min) implying a role of Golgi in the observed Cx43-dependent coherence in collective motility. BFA-treated HeLa 43 showed a change of SD from 0.004–0.005 µm/min, which was more similar to HeLa WT than in the untreated conditions. For confirmation, the percentage area closure was also plotted that showed similar key trends as the velocity of front (Fig. S4). Since coherent motility has been reported to be regulated during collective cell migration involved in neural crest cells undergoing epithelial to mesenchymal transition (EMT) (Carmona-Fontaine et al., 2011), we next verify is HeLa 43 also displays signatures of EMT.

### Cx43 expression is associated with EMT markers’ expression

**Fig. 6.**
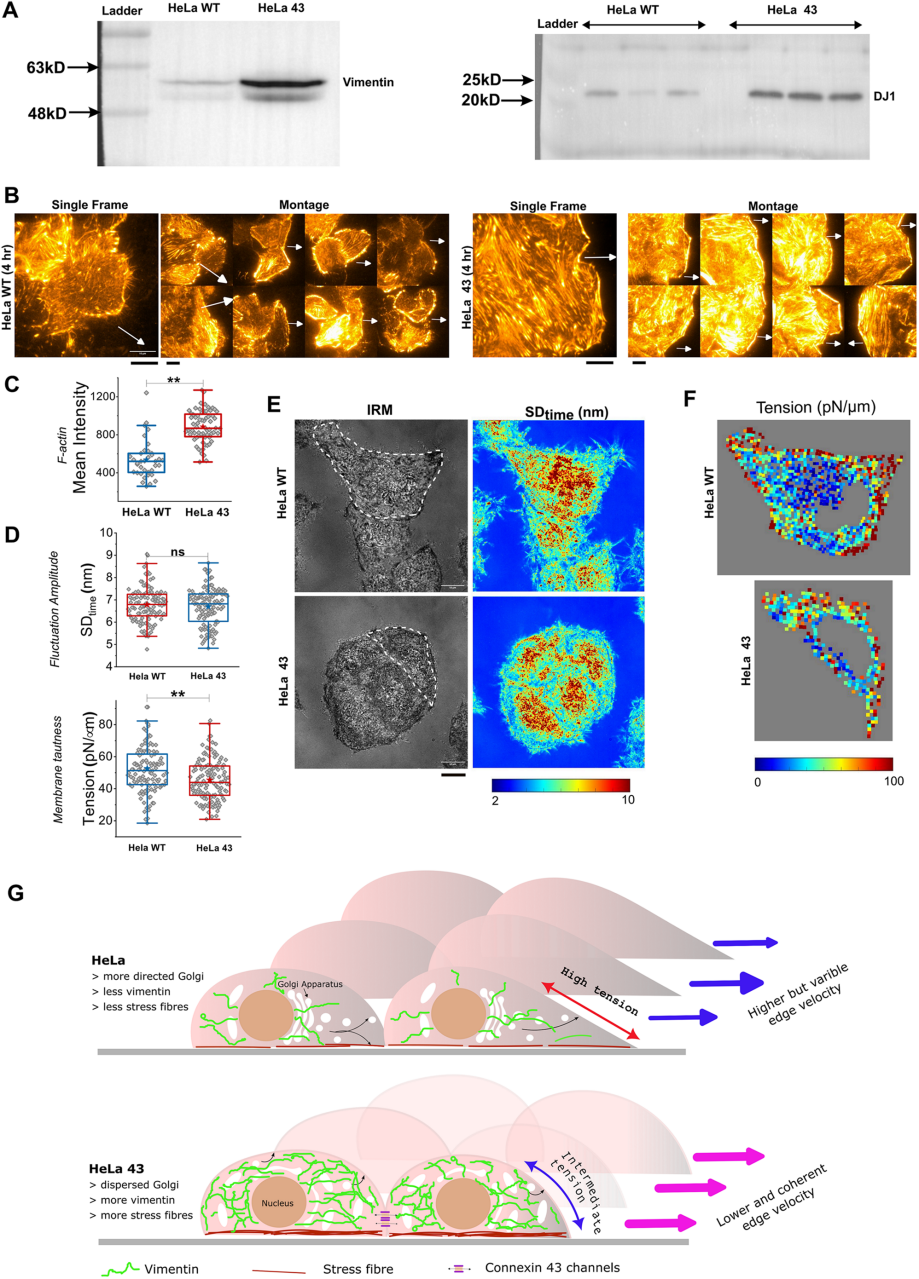
**Mechanical differences between HeLa WT and HeLa 43 cells.** (A) Western blot showing higher abundance of intermediate filament protein vimentin (left blot) and the protein DJ-1 (right blot) in HeLa 43. (B) Representative TIRF images (heat map: light regions: higher concentration) of basal F-actin in HeLa WT and HeLa 43 stained with CellMask Orange Actin tracking stain; scale bars: 10 μm. (C) Quantification of F-actin intensity of cells. Number of cells used: HeLa WT: 37, HeLa 43: 68. Arrows point towards the wound. (D) Quantification of fluctuation amplitude and fluctuation-tension in HeLa WT and HeLa 43 cells; number of cells: HeLa WT: 121, HeLa 43: 135 from four independent experiments. (E) Representative IRM images and maps of fluctuation-amplitude in clumps of HeLa WT and HeLa 43 cells. White dotted lines are guides to the eye marking out cell approximate boundaries used next. (F) Tension map of cells outlined in E. For statistical significance (C,D), the Mann–Whitney *U*-test was performed; ns, *P*>0.05; **P<0.001. (G) Graphic depicts HeLa WT and HeLa 43, highlighting key differences in organelles and their distribution and levels as well as membrane mechanics and speed variation of their fronts.

Cell migration on one hand, is crucial for wound closure and organ development, while on the other hand plays a detrimental role in cancer metastasis carried out by EMT by downregulating some epithelial-specific proteins such as E-cadherin and upregulating the mesenchymal specific proteins such as vimentin. Vimentin, a major constituent of the intermediate filament family of proteins, is ubiquitously expressed in normal mesenchymal cells and is known to maintain cellular integrity and provide resistance against stress. Many studies have also found that DJ-1, an antioxidant protein, is highly expressed in various types of cancer, such as breast cancer, cervical and prostate cancer, and endometrial cancer (Kawate et al., 2017). Moreover, heat shock protein 90 (Hsp90), is also reported to promote EMT by regulating NF-κβ pathway, in various cancer cell lines like nasopharyngeal, renal, ovarian, and colorectal cancer (Chong et al., 2019; Nagaraju et al., 2015). Interestingly, our immunoblot data using anti-vimentin, anti-DJ-1, and anti-Hsp90 antibody suggest that Cx43-expressing HeLa cells are inclined towards mesenchymal trait as they significantly express vimentin and DJ-1 (Fig. 6), and Hsp90 (Fig. S5).

### Cx43 expression is associated with higher basal actin and lower tension

Having established that Cx43 causes a higher expression of intermediate filaments – vimentin, we next measured the levels of basal actin in HeLa WT and HeLa 43 cells. It should be noted that branched actin at lamellipodia exerts pushing forces on the membrane aiding migration, and enhanced stress fibres (contractile filament bundles connecting focal adhesions) can also slow down adhesion. Therefore, it was attractive to know how the speed reduction in HeLa 43 was manifested. On imaging actin in live migrating cells (Fig. 6B,C), we found that HeLa 43 has a much-enhanced network of stress fibres accompanied by a higher mean level of filamentous actin per unit area. This indicates that Cx43 might upregulate pathways that enhance and strengthen stress fibres.

With enhanced stress fibres, it was also visible that the lamellipodia regions were less significant in HeLa 43. Therefore, we sought to understand if the pushing forces on the membrane were also lower. Higher pushing forces are expected to enhance membrane tension. Utilizing IRM, we measured fluctuations and derived fluctuation tension (Fig. 6D-F). We found that HeLa 43 displayed higher fluctuations and lower tension than HeLa WT. We also confirmed using micro-patterning and making cells similar shaped (round) that the tension difference between the two cell types persist (Fig. S6). On mapping out local tension within single cells, it was also clear that the HeLa 43 had less tension gradient while HeLa WT had more prominent higher tension at the leading front.

## DISCUSSION

Cell migration is a fundamental process that not only has a pivotal role in early life when embryogenesis occurs, it is also essential for many physiological functions of the adult organism, e.g. for immune surveillance, angiogenesis and wound healing. Moreover, cell migration plays an important role in pathophysiological processes such as tumour growth and metastasis. Directed migration can be aided by an oriented Golgi while to move as a collection, cells need to maintain cell–cell contacts, e.g. by using connexins. In this work, we addressed the interlink between Golgi's role and Cx43’s role and bring out new insights about key requirements and control of cell migration.

In our study, we found that Cx43 overexpression promotes migration, invasion, and EMT in HeLa 43 cells. In a previous study, it was shown that DJ-1 could promote EMT via activating the Wnt signalling pathway (Jin et al., 2020). During the process of tumour progression, DJ-1 activates the MAPK and AKT/mTOR signalling pathway via suppression of the PTEN gene, thereby promoting proliferation and survival by inhibiting apoptosis followed by invasion and metastasis of tumour cells. In addition to this, DJ-1 also serves as an antioxidant to protect the cancer cells from oxidative stress by oxidizing itself or stabilizing Nrf2, the antioxidant transcriptional regulator (Raninga et al., 2017). However, the evidence of DJ-1-inducing EMT is rare. Taking into consideration that EMT is an important step in the process of cancer invasion and metastasis, we have checked the expression of mesenchymal marker vimentin in Cx43-overexpressed cells keeping HeLa WT cells as a control. We found a significant change in protein expression in the case of HeLa 43 cells compared to the HeLa WT, which essentially indicates that Cx43 can regulate the process of EMT in HeLa cells. Our results have shown that Cx43 can promote the expression of DJ-1, which in turn increases the EMT, which can be validated by looking at the increased expression of vimentin in the presence of overexpressed Cx43 in HeLa cells. To support our hypothesis, we have conducted a migration assay for both the cell lines, HeLa WT and HeLa 43, where we found that HeLa 43 shows an increase in coherence as compared to HeLa WT due to an increase in EMT. The probable cause of the activation of EMT is due to the activation of the Wnt/β-catenin signalling pathway, which plays a fundamental role in determining the cell fate during early embryonic development along with the regulation of proliferation, and survival and apoptosis (Liu et al., 2022). However, mutation of the Wnt pathway can lead to cancer due to the activation of the multifunctional protein DJ-1 (Olivo et al., 2022). Our current experiment focused mainly on the relationship between Cx43, DJ-1 and EMT responsible for causing more migration in HeLa 43 cells however the mechanism of how DJ-1 activates the Wnt pathway by inhibiting β-catenin and thereby promoting EMT was not further investigated. Collectively, these results show that Cx43 can promote cervical cancer cell invasion and migration by inducing EMT. EMT and MET form a crucial factor for phenotypic plasticity during metastasis and offer resistance to therapeutic approaches. Recently it has been discovered that the cells undergoing EMT/MET can attain one or more hybrid epithelial/mesenchymal (E/M) phenotypes. This process is termed partial EMT (Liao et al., 2021). Cells in this phenotype are found to exhibit more aggressive behaviour as compared to those in epithelial or mesenchymal states. In our current study, we have investigated that Cx43 upregulates EMT however, the type of EMT getting upregulated is yet to be studied. We strongly think that Cx43 also plays a crucial role in causing partial EMT, which can be a strong prospect as a continuation of this current study.

Data revealing key differences in the membrane mechanics of the two cell types form the first results on which future work on deciphering the proposed mechanisms may be based. Maps of tension (Fig. 6F) suggest the edges of HeLa WT to display a higher gradient. Imaging leaders have revealed a higher percentage (40%) cells of HeLa WT displaying a clear gradient towards the leading edge than HeLa 43 (20%) cells. The need for Golgi's orientation, we believe could be more in HeLa WT since the high front tension may need to be reduced intermittently to enable actin to sustain its polymerization forces at the front. In HeLa 43, cell–cell connections could contribute strongly to coordinated movement thereby reducing the dependency on front-high tension and Golgi reorientation to maintain direction.

In conclusion, we believe that in the presence of Cx43, the utilization of membrane mechanical gradients to achieve directionality is weaker, while stabilization and continuity of basal actin and cytoplasmic vimentin might ensure slow but coordinated movement of cells at the migrating front (Fig. 6G). Evidently, such migration also is less dependent on the Golgi's reorientation as the Golgi, perhaps due to the altered and augmented cytoskeleton is more dispersed and closer to the plasma membrane.

## MATERIALS AND METHODS

### Cells and cell lines

HeLa WT cells obtained from ATCC and maintained in Dulbecco's Modified Eagle Medium supplemented with 10% fetal bovine serum (Invitrogen) and 1% penicillin-streptomycin (Gibco). HeLa WT cell line stably expressing Cx43, referred to as HeLa 43, were generated as previously described (Das Sarma et al., 2002). HeLa 43 cells were maintained in the same medium mentioned above with additional supplementation of G418 (Sigma, A1720) at a final concentration of 500 µg/ml.

### Lucifer yellow dye transfer

The gap junction functional coupling between HeLa WT and HeLa 43 cells was determined using scrape loading of Lucifer Yellow (LY) in confluent monolayers of HeLa WT and HeLa 43 as described previously (Li et al., 2005), with minor modifications. HeLa WT and HeLa 43 were scrapes loaded with PBS containing 4 mg/ml LY CH (Sigma, Saint Louis, MO, USA). After a 1-min incubation, the LY solution was removed from the culture, the culture was washed thoroughly with PBS, and respective fresh culture medium was added. The distance of LY spread from the scrape-loading point to neighbouring cells was imaged using an inverted microscope with a Hamamatsu Orca-1 charge-coupled device (CCD) camera, and the distance spread was measured using ImageJ software.

### Cell migration assay

Scratches were created on a confluent monolayer of HeLa WT and HeLa 43 with thorough washing with PBS to remove non-adherent cells, after which cell-specific media was added. For disruption of proteins translocation from ER to Golgi, Brefeldin A (B6542, Sigma), a known disruptor of structure and functions of Golgi, was added to a final concentration of 0.2 μg/ml in the culture media. Cells were monitored for migration capacity at 0, 4, 8 and 12 h using a bright field microscope. The culture medium was supplemented with 0.2 µM of Hoechst solution for immunofluorescence studies. Results were analysed using ImageJ software and interpreted as described in supplementary materials.

### Analysis of front velocity

ROIs were drawn around scratch region following the cell edges such that their area (A) represented the area of the section of wound. The length (L) along the wound that each ROI covered was kept constant for all ROIs across conditions and time points. With time, as the cell edges would move closer, redrawn ROIs would capture the reduction in area (Δ***A***(***t***)=***A***(***t***)−***A***(***t***+Δ***t***)). Since the area could be measured most robustly, area change was used to derive the effective average velocity from ROIs. At any time point, since the length of the ROIs were L, the representative rectangle having same length L and same area ***A***(***t***), would have a breadth (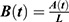). This breadth ***B***(***t***) is representative of the mean edge to edge distance. Lowering of ***B***(***t***) with time (Δ***B***(***t***)=***B***(***t***)−***B***(***t***+Δ***t***)) can be imagined to be due to movement of two front towards each other. Again, averaging over the two, the mean distance covered by the front would be (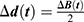) – which divided by the time interval (Δ***t***) would give the average velocity of the front (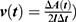. Multiple ROIs and scratches over different trials were used for comparisons.

### Immunofluorescences

Immunofluorescence studies were done according to a protocol described previously (Das Sarma et al., 2001), with minor modifications. For standard immunofluorescence, Cells were plated on etched glass coverslips and fixed with 4% paraformaldehyde (PFA). Permeabilization was done with phosphate-buffered saline (PBS) containing 0.5% Triton X-100 and blocked with blocking solution [PBS containing 0.5% Triton X-100 and 2.5% heat-inactivated goat serum (PBS-GS)]. The cells were incubated with anti-Golgin 97 (Invitrogen, 14-9767-82) (1:500), anti-Cx43 (Sigma, C6219) (1:1000), anti-GM130 (BD Biosciences, 610822) (1:500) antibodies diluted in a blocking solution for 1 h, washed, and then labelled with secondary antibodies conjugated with Alexa fluor 568 (Invitrogen, A10042) (1:1000), Alexa fluor 488 (Invitrogen, A11001) (1:1000) diluted in a blocking solution. Then cells were visualized using a Zeiss confocal microscope (LSM710). Images were acquired and processed with Zen2010 software (Carl Zeiss).

### Membrane protein isolation and analysis

Monolayer of HeLa WT and HeLa 43 cells were washed, then harvested in PBS containing protease inhibitor cocktail (complete protease inhibitor; Roche, Mannheim, Germany) and phosphatase inhibitors (1 mM NaVO_4_ and 10 mM NaF), and then passed through a Dounce homogenizer 100 times (Koval et al., 1997). The homogenate was centrifuged at 500 ***g*** for 5 min using an Eppendorf 5415 R centrifuge. The resulting supernatant was centrifuged at 100,000 ***g*** for 30 min using a Beckman Optima Max ultracentrifuge to obtain a membrane-enriched pellet. To analyse total membrane connexin expression, this pellet was resuspended in RIPA buffer and subjected to immunoblot analysis for Cx43.

For detergent solubilization studies, the membrane-enriched pellet was resuspended in PBS with protease and phosphatase inhibitors at 4°C containing 1% Triton X-100 and then incubated for 30 min at 4°C. Care was taken to ensure that the samples were not warmed to room temperature (RT) during extraction. The sample was centrifuged at 100,000 ***g*** for 30 min and separated into Triton X-100 soluble supernatant and insoluble pellet fractions (Musil and Goodenough, 1991). The soluble fraction was then diluted into RIPA buffer, while the insoluble fraction was initially resuspended in PBS with 1% Triton X-100 before dilution. Equal volumes of soluble and insoluble fractions were probed for Cx43 by immunoblotting.

### Immunoblot analysis

For immunoblotting, 15 µg protein was loaded per well. Subsequently, samples were resolved by SDS-PAGE using a 12% polyacrylamide gel, transferred to polyvinylidene difluoride (PVDF) membranes using transfer buffer (25 mM Tris, 192 mM glycine, and 20% methanol), and blocked for 1 h at RT using blocking solution (5%, wt/vol, powdered milk dissolved in TBST or Tris-buffered saline containing 0.1%, vol/vol, Tween 20). The samples were then incubated overnight at 4°C using anti-Cx43 (Sigma, C6219) (1:5000), anti-DJ-1 (home made serum antibody) (1:500), anti-vimentin (CST, 5741) (1:1000), anti-Hsp90 (Biobharti, BBAB021) (1:1000) diluted in blocking solution, followed by washes with TBST and then a 1-h incubation of horseradish peroxidase (HRP)-conjugated secondary IgG in blocking solution. The immunoblots were washed in TBST, and then immunoreactive bands were visualized using Super Signal WestPico chemiluminescent substrate (Thermo Scientific, Rockford, IL, USA). Densitometric analysis of non-saturated films was performed using Bio-Rad Quantity One analysis software (Hercules, CA, USA) or using a Syngene G: Box ChemiDoc system and GENESys software.

### Interference reflection microscopy

For obtaining membrane fluctuation and fluctuation tension, interference reflection microscopy (IRM) was performed in an onstage 37°C incubator (Tokai Hit, Japan). An inverted microscope (Nikon, Japan) equipped with a 100 W mercury arc lamp, an interference filter (546±12 nm), a 50-50 beam splitter, a 60X water-immersion objective (1.22 NA), and an sCMOS camera (Hamamatsu, Japan) was used. Imaging was performed with 50 ms exposure time capturing a total of 2048 frames for any single measurement.

### Total internal reflection fluorescence microscopy

For assessing basal actin in migrating cells using TIRF, cells were grown into a monolayer and were scratched to create a wound-like region. Cells were subsequently incubated for 3 h and 45 min, followed by applying CellMask Orange Actin tracking stain (Invitrogen, USA) at a 0.5 µM. Cells were imaged after 15 min of incubation in the live actin stain.

Imaging was performed using an inverted microscope (Olympus, Japan) with a 100x 1.49 NA oil immersion TIRF objective using a 561 nm laser. The exposure time of 200 ms and a final pixel size of 65 nm was maintained throughout.

### Quantification and statistical analysis

Calibration and analysis of IRM were performed as described previously (Biswas et al., 2017). Briefly, beads (60 µm diameter polystyrene beads; Bangs laboratories) were imaged on each day of experiments to find the intensity to conversion factor after comparison with IRM images of cells. Subsequently, pixel, where this conversion held, were identified and termed first branch regions (FBRs). Groups of 12×12 pixels (0.89×0.89 µm^2^) were used to sample single cells locally to recreate migrating cells’ tension landscape. Measurements of fluctuations only from FBRs were used for quantitative analysis. The standard deviation (SD) of relative heights (of 2048 frames) calculated at every pixel of an FBR (144 pixels) was averaged to find the SD_time_. To find the tension (fluctuation tension), the power spectral density of the fluctuations was obtained using MATLAB's covariance method (autoregressive PSD estimation) and fitted with the Helfrich-based theoretical model also used earlier.

## Supplementary Material

10.1242/biolopen.060006_sup1 Supplementary information

## References

[BIO060006C1] Arima, S., Nishiyama, K., Ko, T., Arima, Y., Hakozaki, Y., Sugihara, K., Koseki, H., Uchijima, Y., Kurihara, Y. and Kurihara, H. (2011). Angiogenic morphogenesis driven by dynamic and heterogeneous collective endothelial cell movement. *Development* 138, 4763-4776. 10.1242/dev.068023 21965612

[BIO060006C2] Astin, J. W., Batson, J., Kadir, S., Charlet, J., Persad, R. A., Gillatt, D., Oxley, J. D. and Nobes, C. D. (2010). Competition amongst Eph receptors regulates contact inhibition of locomotion and invasiveness in prostate cancer cells. *Nat. Cell Biol.* 12, 1194-1204. 10.1038/ncb2122 21076414

[BIO060006C3] Atia, L., Fredberg, J. J., Gov, N. S. and Pegoraro, A. F. (2021). Are cell jamming and unjamming essential in tissue development? *Cells Dev.* 168, 203727. 10.1016/j.cdev.2021.203727 34363993 PMC8935248

[BIO060006C4] Barriga, E. H., Maxwell, P. H., Reyes, A. E. and Mayor, R. (2013). The hypoxia factor Hif-1α controls neural crest chemotaxis and epithelial to mesenchymal transition. *J. Cell Biol.* 201, 759-776. 10.1083/jcb.201212100 23712262 PMC3664719

[BIO060006C5] Batson, J., Maccarthy-Morrogh, L., Archer, A., Tanton, H. and Nobes, C. D. (2014). EphA receptors regulate prostate cancer cell dissemination through Vav2-RhoA mediated cell-cell repulsion. *Biol. Open* 3, 453-462. 10.1242/bio.20146601 24795148 PMC4058079

[BIO060006C6] Becker, S. F. S., Mayor, R. and Kashef, J. (2013). Cadherin-11 mediates contact inhibition of locomotion during Xenopus neural crest cell migration. *PLoS ONE* 8, e85717. 10.1371/journal.pone.0085717 24392028 PMC3877381

[BIO060006C7] Bindschadler, M. and McGrath, J. L. (2007). Sheet migration by wounded monolayers as an emergent property of single-cell dynamics. *J. Cell Sci.* 120, 876-884. 10.1242/jcs.03395 17298977

[BIO060006C8] Biswas, A., Alex, A. and Sinha, B. (2017). Mapping Cell Membrane Fluctuations Reveals Their Active Regulation and Transient Heterogeneities. *Biophys. J.* 113, 1768-1781. 10.1016/j.bpj.2017.08.041 29045871 PMC5647594

[BIO060006C9] Carmona-Fontaine, C., Matthews, H. and Mayor, R. (2008). Directional cell migration in vivo: Wnt at the crest. *Cell Adh. Migr.* 2, 240-242. 10.4161/cam.2.4.6747 19262160 PMC2633683

[BIO060006C10] Carmona-Fontaine, C., Theveneau, E., Tzekou, A., Tada, M., Woods, M., Page, K. M., Parsons, M., Lambris, J. D. and Mayor, R. (2011). Complement fragment C3a controls mutual cell attraction during collective cell migration. *Dev. Cell* 21, 1026-1037. 10.1016/j.devcel.2011.10.012 22118769 PMC3272547

[BIO060006C11] Chakraborty, M., Mukherjee, B., Nalinakshan, N., Biswas, A., Nayak, R. K. and Sinha, B. (2022). Effect of heterogeneous substrate adhesivity of follower cells on speed and tension profile of leader cells in primary keratocyte collective cell migration. *Biol. Open* 11, bio058893. 10.1242/bio.058893 35146504 PMC8918985

[BIO060006C12] Chong, K. Y., Kang, M., Garofalo, F., Ueno, D., Liang, H., Cady, S., Madarikan, O., Pitruzzello, N., Tsai, C.-H., Hartwich, T. M. P. et al. (2019). Inhibition of Heat Shock Protein 90 suppresses TWIST1 Transcription. *Mol. Pharmacol.* 96, 168-179. 10.1124/mol.119.116137 31175180

[BIO060006C13] Das Sarma, J., Meyer, R. A., Wang, F., Abraham, V., Lo, C. W. and Koval, M. (2001). Multimeric connexin interactions prior to the trans-Golgi network. *J. Cell Sci.* 114, 4013-4024. 10.1242/jcs.114.22.4013 11739633

[BIO060006C14] Das Sarma, J., Wang, F. and Koval, M. (2002). Targeted gap junction protein constructs reveal connexin-specific differences in oligomerization. *J. Biol. Chem.* 277, 20911-20918. 10.1074/jbc.M111498200 11929864

[BIO060006C15] Devreotes, P. and Horwitz, A. R. (2015). Signaling networks that regulate cell migration. *Cold Spring Harb. Perspect Biol.* 7, a005959. 10.1101/cshperspect.a005959 26238352 PMC4526752

[BIO060006C16] Epifantseva, I. and Shaw, R. M. (2018). Intracellular trafficking pathways of Cx43 gap junction channels. *Biochim. Biophys. Acta Biomembr.* 1860, 40-47. 10.1016/j.bbamem.2017.05.018 28576298 PMC5731482

[BIO060006C17] Etienne-Manneville, S. and Hall, A. (2001). Integrin-mediated activation of Cdc42 controls cell polarity in migrating astrocytes through PKCzeta. *Cell* 106, 489-498. 10.1016/S0092-8674(01)00471-8 11525734

[BIO060006C18] Francis, R., Xu, X., Park, H., Wei, C.-J., Chang, S., Chatterjee, B. and Lo, C. (2011). Connexin43 modulates cell polarity and directional cell migration by regulating microtubule dynamics. *PLoS ONE* 6, e26379. 10.1371/journal.pone.0026379 22022608 PMC3194834

[BIO060006C19] Friedl, P. and Gilmour, D. (2009). Collective cell migration in morphogenesis, regeneration and cancer. *Nat. Rev. Mol. Cell Biol.* 10, 445-457. 10.1038/nrm2720 19546857

[BIO060006C20] Gauthier, N. C., Fardin, M. A., Roca-Cusachs, P. and Sheetz, M. P. (2011). Temporary increase in plasma membrane tension coordinates the activation of exocytosis and contraction during cell spreading. *Proc. Natl Acad. Sci. USA* 108, 14467-14472. 10.1073/pnas.1105845108 21808040 PMC3167546

[BIO060006C21] Goodenough, D. A. and Paul, D. L. (2009). Gap junctions. *Cold Spring Harb. Perspect Biol.* 1, a002576. 10.1101/cshperspect.a002576 20066080 PMC2742079

[BIO060006C22] Gourdie, R. G., Ghatnekar, G. S., O'Quinn, M., Rhett, M. J., Barker, R. J., Zhu, C., Jourdan, J. and Hunter, A. W. (2006). The unstoppable connexin43 carboxyl-terminus: new roles in gap junction organization and wound healing. *Ann. N. Y. Acad. Sci.* 1080, 49-62. 10.1196/annals.1380.005 17132774

[BIO060006C23] Hsu, R.-M., Zhong, C.-Y., Wang, C.-L., Liao, W.-C., Yang, C., Lin, S.-Y., Lin, J.-W., Cheng, H.-Y., Li, P.-Y. and Yu, C.-J. ( 2018). Golgi tethering factor golgin-97 suppresses breast cancer cell invasiveness by modulating NF-κB activity. *Cell Commun. Signal.* 16, 19. 10.1186/s12964-018-0230-5 29703230 PMC5923015

[BIO060006C24] Jakobsson, L., Franco, C. A., Bentley, K., Collins, R. T., Ponsioen, B., Aspalter, I. M., Rosewell, I., Busse, M., Thurston, G., Medvinsky, A. et al. (2010). Endothelial cells dynamically compete for the tip cell position during angiogenic sprouting. *Nat. Cell Biol.* 12, 943-953. 10.1038/ncb2103 20871601

[BIO060006C25] Jin, F., Wang, H., Li, D., Fang, C., Li, W., Shi, Q., Diao, Y., Ding, Z., Dai, X., Tao, L. et al. (2020). DJ-1 promotes cell proliferation and tumor metastasis in esophageal squamous cell carcinoma via the Wnt/β-catenin signaling pathway. *Int. J. Oncol.* 56, 1115-1128. 32319588 10.3892/ijo.2020.5005 PMC7115355

[BIO060006C26] Kawate, T., Tsuchiya, B. and Iwaya, K. (2017). Expression of DJ-1 in cancer cells: its correlation with clinical significance. *Adv. Exp. Med. Biol.* 1037, 45-59. 10.1007/978-981-10-6583-5_4 29147902

[BIO060006C27] Keren, K., Pincus, Z., Allen, G. M., Barnhart, E. L., Marriott, G., Mogilner, A. and Theriot, J. A. (2008). Mechanism of shape determination in motile cells. *Nature* 453, 475-480. 10.1038/nature06952 18497816 PMC2877812

[BIO060006C28] Koval, M., Harley, J. E., Hick, E. and Steinberg, T. H. (1997). Connexin46 is retained as monomers in a trans-golgi compartment of osteoblastic cells. *J. Cell Biol.* 137, 847-857. 10.1083/jcb.137.4.847 9151687 PMC2139843

[BIO060006C29] Lebreton, G. and Casanova, J. (2014). Specification of leading and trailing cell features during collective migration in the Drosophila trachea. *J. Cell Sci.* 127, 465-474. 24213534 10.1242/jcs.142737

[BIO060006C30] Li, W., Hertzberg, E. L. and Spray, D. C. (2005). Regulation of Connexin43-Protein Binding in Astrocytes in Response to Chemical Ischemia/Hypoxia. *J. Biol. Chem.* 280, 7941-7948. 10.1074/jbc.M410548200 15618229

[BIO060006C31] Liao, C., Wang, Q., An, J., Long, Q., Wang, H., Xiang, M., Xiang, M., Zhao, Y., Liu, Y., Liu, J. et al. (2021). Partial EMT in squamous cell carcinoma: a snapshot. *Int. J. Biol. Sci.* 17, 3036-3047. 10.7150/ijbs.61566 34421348 PMC8375241

[BIO060006C32] Lieber, A. D., Schweitzer, Y., Kozlov, M. M. and Keren, K. (2015). Front-to-rear membrane tension gradient in rapidly moving cells. *Biophys. J.* 108, 1599-1603. 10.1016/j.bpj.2015.02.007 25863051 PMC4390806

[BIO060006C33] Liu, J., Xiao, Q., Xiao, J., Niu, C., Li, Y., Zhang, X., Zhou, Z., Shu, G. and Yin, G. (2022). Wnt/β-catenin signalling: function, biological mechanisms, and therapeutic opportunities. *Signal Transduct. Target. Ther.* 7, 3. 10.1038/s41392-021-00762-6 34980884 PMC8724284

[BIO060006C34] Matthews, H. K., Marchant, L., Carmona-Fontaine, C., Kuriyama, S., Larraín, J., Holt, M. R., Parsons, M. and Mayor, R. (2008). Directional migration of neural crest cells in vivo is regulated by Syndecan-4/Rac1 and non-canonical Wnt signaling/RhoA. *Development* 135, 1771-1780. 10.1242/dev.017350 18403410

[BIO060006C35] Mayor, R. and Theveneau, E. (2014). The role of the non-canonical Wnt-planar cell polarity pathway in neural crest migration. *Biochem. J.* 457, 19-26. 10.1042/BJ20131182 24325550

[BIO060006C36] Mimori-Kiyosue, Y., Grigoriev, I., Lansbergen, G., Sasaki, H., Matsui, C., Severin, F., Galjart, N., Grosveld, F., Vorobjev, I., Tsukita, S. et al. (2005). CLASP1 and CLASP2 bind to EB1 and regulate microtubule plus-end dynamics at the cell cortex. *J. Cell Biol.* 168, 141-153. 10.1083/jcb.200405094 15631994 PMC2171674

[BIO060006C37] Musil, L. S. and Goodenough, D. A. (1991). Biochemical analysis of connexin43 intracellular transport, phosphorylation, and assembly into gap junctional plaques. *J. Cell Biol.* 115, 1357-1374. 10.1083/jcb.115.5.1357 1659577 PMC2289231

[BIO060006C38] Nagaraju, G. P., Long, T.-E., Park, W., Landry, J. C., Taliaferro-Smith, L., Farris, A. B., Diaz, R. and El-Rayes, B. F. (2015). Heat shock protein 90 promotes epithelial to mesenchymal transition, invasion, and migration in colorectal cancer. *Mol. Carcinog.* 54, 1147-1158. 10.1002/mc.22185 24861206

[BIO060006C39] Natividad, R. J., Lalli, M. L., Muthuswamy, S. K. and Asthagiri, A. R. (2018). Golgi Stabilization, not its front-rear bias, is associated with EMT-enhanced fibrillar migration. *Biophys. J.* 115, 2067-2077. 10.1016/j.bpj.2018.10.006 30366626 PMC6343588

[BIO060006C40] Ofer, N., Mogilner, A. and Keren, K. (2011). Actin disassembly clock determines shape and speed of lamellipodial fragments. *Proc. Natl Acad. Sci. USA* 108, 20394-20399. 10.1073/pnas.1105333108 22159033 PMC3251093

[BIO060006C41] Olivo, E., La Chimia, M., Ceramella, J., Catalano, A., Chiaradonna, F., Sinicropi, M. S., Cuda, G., Iacopetta, D. and Scumaci, D. (2022). Moving beyond the Tip of the Iceberg: DJ-1 Implications in cancer metabolism. *Cells* 11, 1432. 10.3390/cells11091432 35563738 PMC9103122

[BIO060006C42] Palazzo, A. F., Joseph, H. L., Chen, Y.-J., Dujardin, D. L., Alberts, A. S., Pfister, K. K., Vallee, R. B. and Gundersen, G. G. (2001). Cdc42, dynein, and dynactin regulate MTOC reorientation independent of Rho-regulated microtubule stabilization. *Curr. Biol.* 11, 1536-1541. 10.1016/S0960-9822(01)00475-4 11591323

[BIO060006C43] Park, J.-A., Kim, J. H., Bi, D., Mitchel, J. A., Qazvini, N. T., Tantisira, K., Park, C. Y., McGill, M., Kim, S.-H., Gweon, B. et al. (2015). Unjamming and cell shape in the asthmatic airway epithelium. *Nat. Mater.* 14, 1040-1048. 10.1038/nmat4357 26237129 PMC4666305

[BIO060006C44] Piroli, M. E., Blanchette, J. O. and Jabbarzadeh, E. (2019). Polarity as a physiological modulator of cell function. *Front. Biosci. (Landmark Ed)* 24, 451-462. 10.2741/4728 30468666 PMC6343491

[BIO060006C45] Qasba, P. K., Ramakrishnan, B. and Boeggeman, E. (2008). Structure and function of beta −1,4-galactosyltransferase. *Curr. Drug Targets* 9, 292-309. 10.2174/138945008783954943 18393823 PMC2365515

[BIO060006C46] Raninga, P. V., Di Trapani, G. and Tonissen, K. F. (2017). The multifaceted roles of DJ-1 as an antioxidant. *Adv. Exp. Med. Biol.* 1037, 67-87. 10.1007/978-981-10-6583-5_6 29147904

[BIO060006C47] Reig, G., Pulgar, E. and Concha, M. L. (2014). Cell migration: from tissue culture to embryos. *Development* 141, 1999-2013. 10.1242/dev.101451 24803649

[BIO060006C48] Rørth, P. (2009). Collective cell migration. *Annu. Rev. Cell Dev. Biol.* 25, 407-429. 10.1146/annurev.cellbio.042308.113231 19575657

[BIO060006C49] Shnitsar, I. and Borchers, A. (2008). PTK7 recruits dsh to regulate neural crest migration. *Development* 135, 4015-4024. 10.1242/dev.023556 19004858

[BIO060006C50] Solan, J. L. and Lampe, P. D. (2005). Connexin phosphorylation as a regulatory event linked to gap junction channel assembly. *Biochim. Biophys. Acta* 1711, 154-163. 10.1016/j.bbamem.2004.09.013 15955300

[BIO060006C51] Theveneau, E., Marchant, L., Kuriyama, S., Gull, M., Moepps, B., Parsons, M. and Mayor, R. (2010). Collective chemotaxis requires contact-dependent cell polarity. *Dev. Cell* 19, 39-53. 10.1016/j.devcel.2010.06.012 20643349 PMC2913244

[BIO060006C52] Villar-Cerviño, V., Molano-Mazón, M., Catchpole, T., Valdeolmillos, M., Henkemeyer, M., Martínez, L. M., Borrell, V. and Marín, O. (2013). Contact repulsion controls the dispersion and final distribution of Cajal-Retzius cells. *Neuron* 77, 457-471. 10.1016/j.neuron.2012.11.023 23395373 PMC3569744

[BIO060006C53] Vitorino, P. and Meyer, T. (2008). Modular control of endothelial sheet migration. *Genes Dev.* 22, 3268-3281. 10.1101/gad.1725808 19056882 PMC2600767

[BIO060006C54] Yadav, S. and Linstedt, A. D. (2011). Golgi positioning. *Cold Spring Harb. Perspect Biol.* 3, a005322. 10.1101/cshperspect.a005322 21504874 PMC3101843

[BIO060006C55] Yadav, S., Puri, S. and Linstedt, A. D. (2009). A primary role for Golgi positioning in directed secretion, cell polarity, and wound healing. *Mol. Biol. Cell* 20, 1728-1736. 10.1091/mbc.e08-10-1077 19158377 PMC2655245

[BIO060006C56] Zeng, J., Feng, S., Wu, B. and Guo, W. (2017). Polarized exocytosis. *Cold Spring Harb. Perspect Biol.* 9, a027870. 10.1101/cshperspect.a027870 28246185 PMC5710101

[BIO060006C57] Zhang, X.-F. and Cui, X. (2017). Connexin 43: Key roles in the skin. *Biomed. Rep.* 6, 605-611. 10.3892/br.2017.903 28584630 PMC5449964

