# Supplementary material for: Connexin 43 mediated collective cell migration is independent of Golgi orientation
Source: Biol Open. 2023 Oct 30;12(10):bio060006. doi: 10.1242/bio.060006 (PMC10629497; doi:10.1242/bio.060006)
Supplement: Supplementary information [file biolopen-12-060006-s1.pdf]

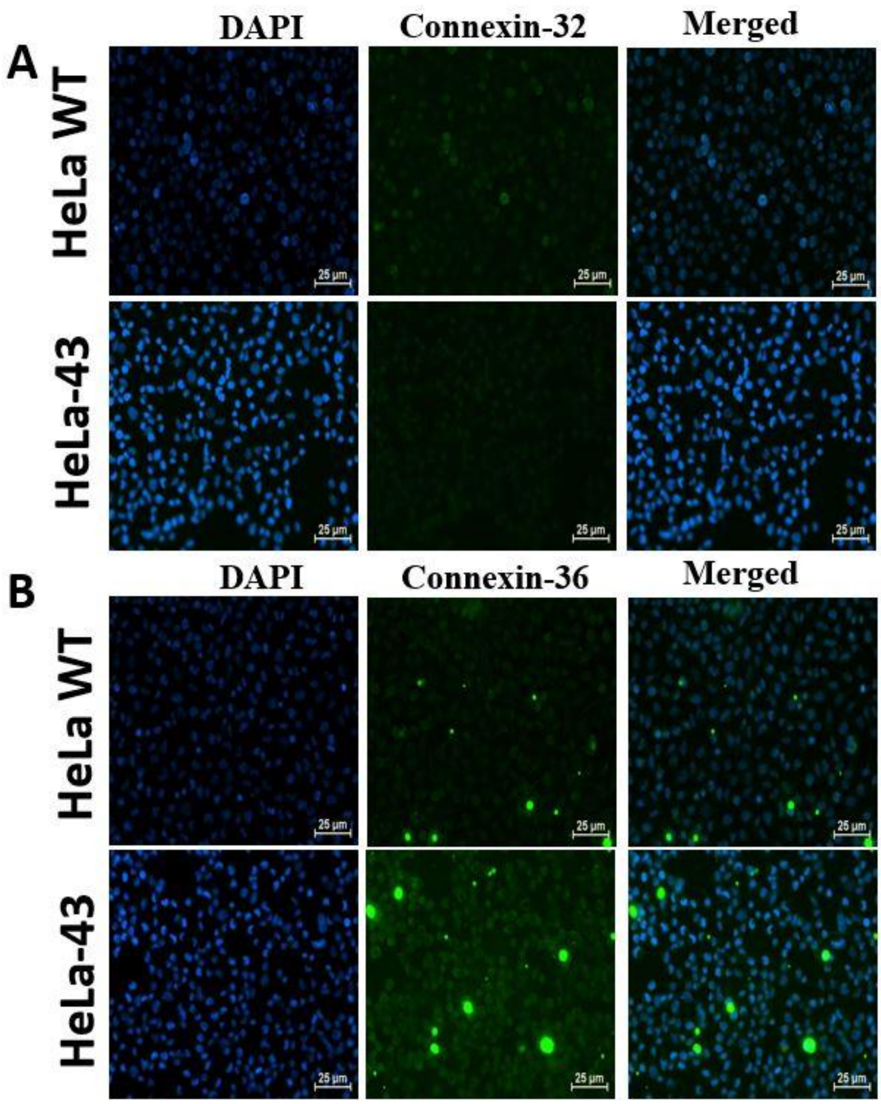

Fig. S1. Hela WT and Hela 43 do not express Connexin 32 and 36.

Epi-fluorescence images of HeLa WT and HeLa 43 shows no expression of Connexin 32 (green)(A). Immunofluorescence analysis shows that HeLa WT and HeLa 43 do not express Connexin 36 (Green) (B). The scale bar is 25 µm.

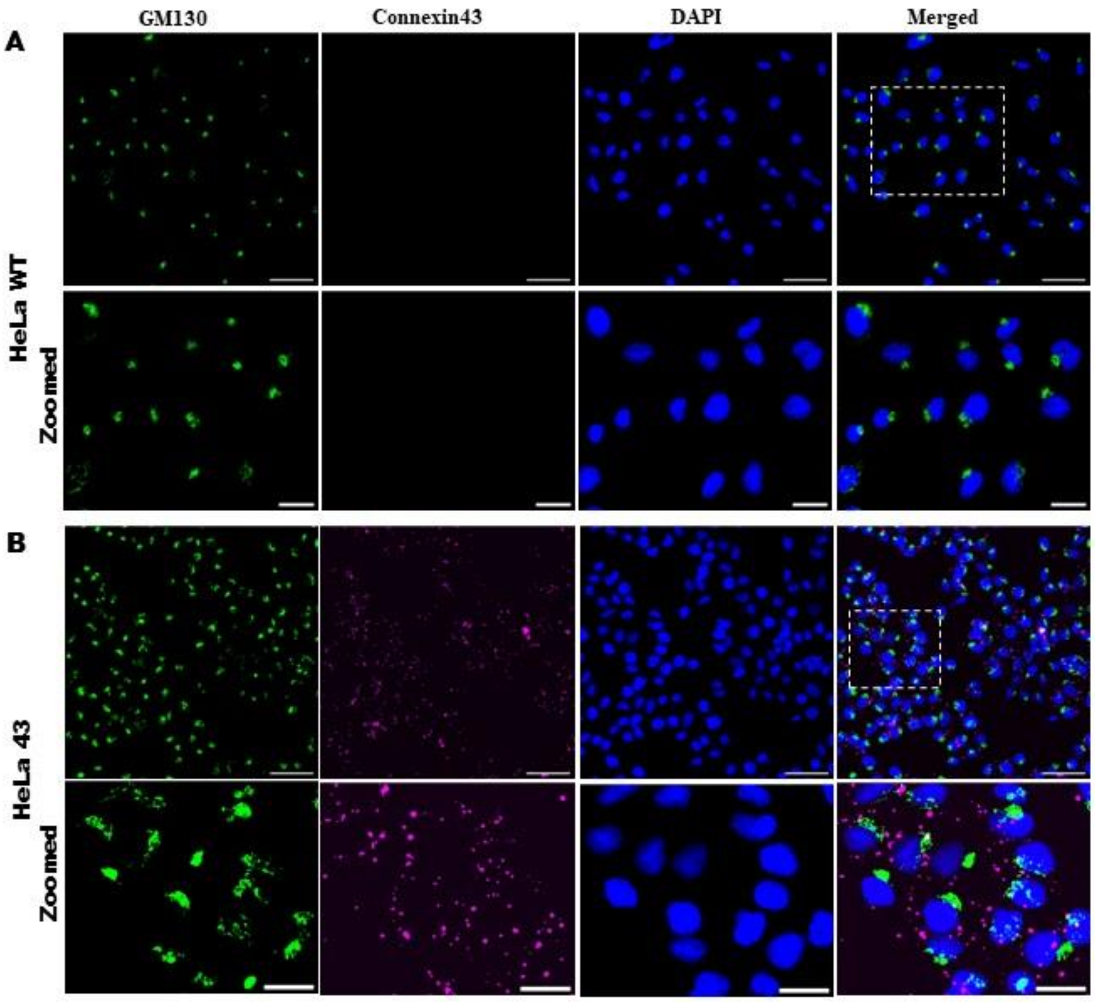

Fig. S2. Characterizing HeLa WT and HeLa 43 using Cis Golgi protein GM130. Epi-fluorescence images of HeLa WT (A) and HeLa 43 (B) highlighting Cis Golgi and Connexin 43 distribution. Both cell types have condensed cis-Golgi (Green) whereases Hela 43 express Connexin 43 (Magenta). Scale bar is 50µm and for Zoomed images 20µm.

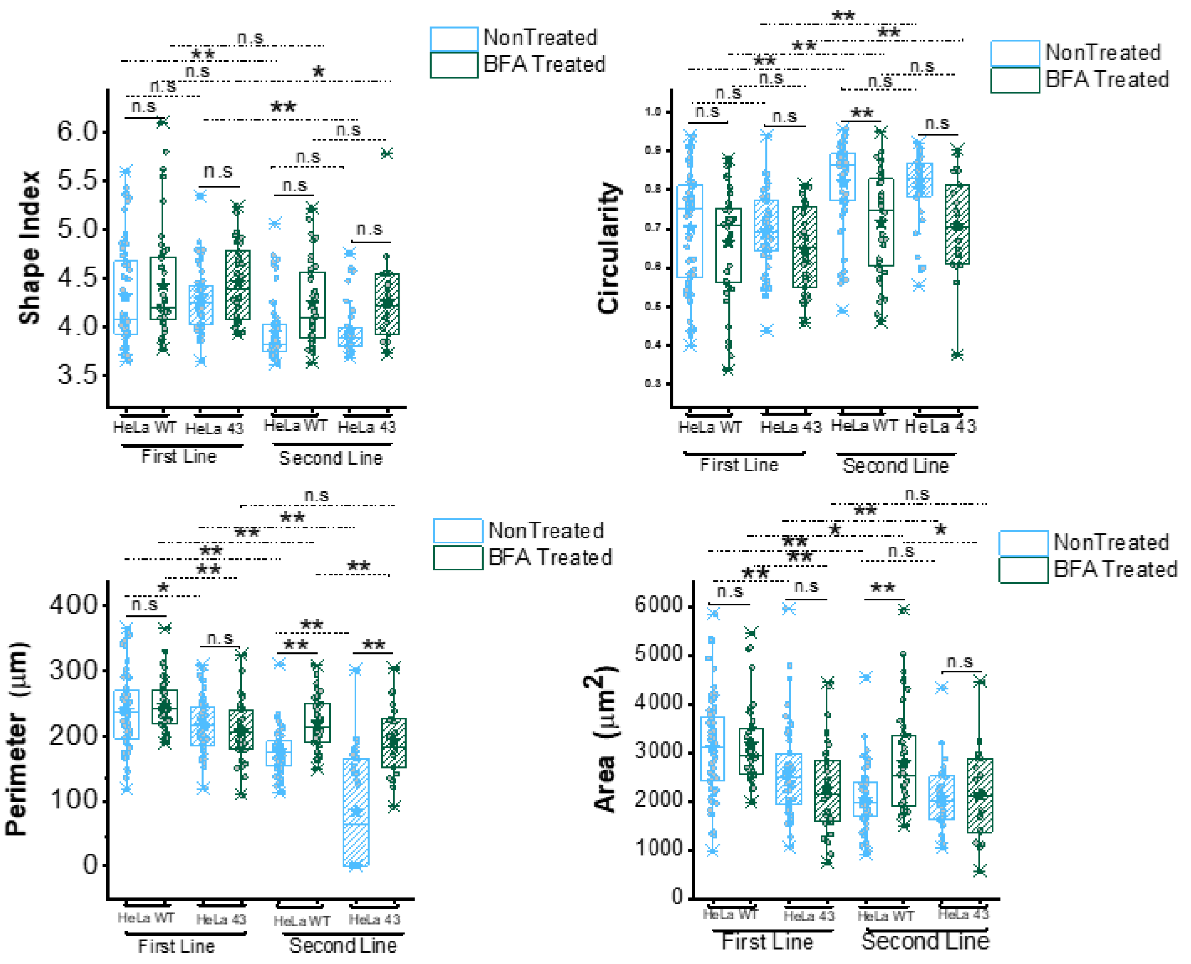

Fig. S3. Comparison of other single cell parameters. Single cell parameters obtained from scratch assays on monolayers after 12 hrs of migration post-wounding. For statistical significance, the Mann-Whitney U test was performed, * denotes p values < 0.05, and ** denotes p value < 0.01. Representative of two independent experiments. N = 50 regions (of scratch assays).

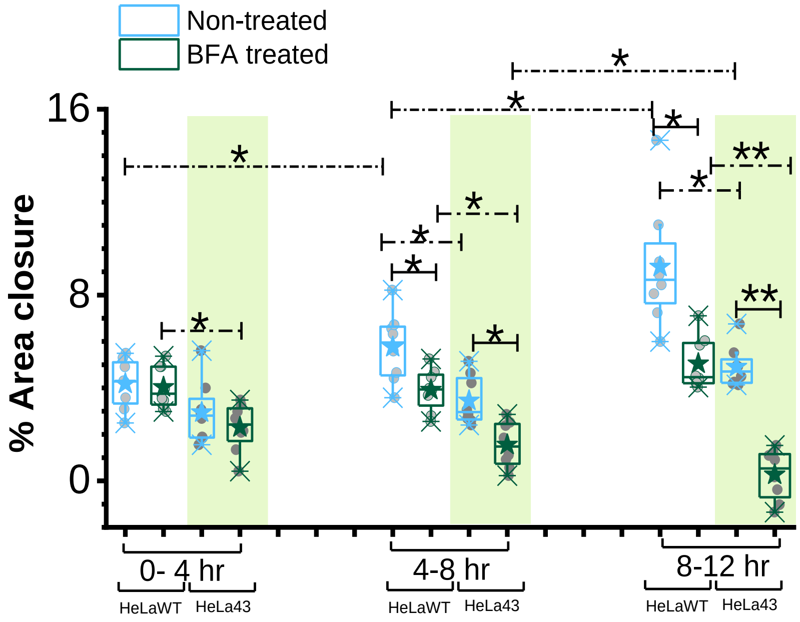

			HeLa WT Control 0-4 hr	HeLa WT Control 4-8 hr	HeLa WT Control 8-12 hr	HeLa WT BFA 0-4 hr	HeLa WT BFA 4-8 hr	HeLa WT BFA 8-12 hr	HeLa 43 Control 0-4 hr	HeLa 43 Control 4-8 hr	HeLa 43 Control 8-12 hr	HeLa 43 BFA 0-4 hr	HeLa 43 BFA 4-8 hr	HeLa 43 BFA 8-12 hr
HeLa WT	Control	0-4 hr	1.000	0.028	0.000	0.798	0.645	0.279	0.065	0.161	0.328	0.005	0.001	0.000
HeLa WT	Control	4-8 hr		1.000	0.005	0.021	0.028	0.279	0.003	0.005	0.279	0.000	0.000	0.000
HeLa WT	Control	8-12 hr			1.000	0.000	0.000	0.001	0.000	0.000	0.000	0.000	0.000	0.000
HeLa WT	BFA	0-4 hr				1.000	0.878	0.083	0.065	0.161	0.065	0.003	0.000	0.000
HeLa WT	BFA	4-8 hr					1.000	0.065	0.161	0.442	0.050	0.007	0.001	0.000
HeLa WT	BFA	8-12 hr						1.000	0.003	0.050	0.959	0.000	0.000	0.000
HeLa 43	Control	0-4 hr							1.000	0.505	0.007	0.645	0.028	0.000
HeLa 43	Control	4-8 hr								1.000	0.038	0.130	0.002	0.000
HeLa 43	Control	8-12 hr									1.000	0.000	0.000	0.000
HeLa 43	BFA	0-4 hr											0.161	0.003
HeLa 43	BFA	4-8 hr											1.000	0.050
HeLa 43	BFA	8-12 hr												1.000

Fig. S4. Comparison of percentage wound closure. Top: Comparison of percentage area closure ($\frac{Area_t - Area_{t+\Delta t}}{\Delta t} . 100, where\ \Delta t = 4\ hr$) for scratch assay of HeLa and HeLa 43 cells as depicted in main **Fig. 5 C**. Bottom panel shows the P-values calculated using Mann Whitney test.

Biology Open • Supplementary information

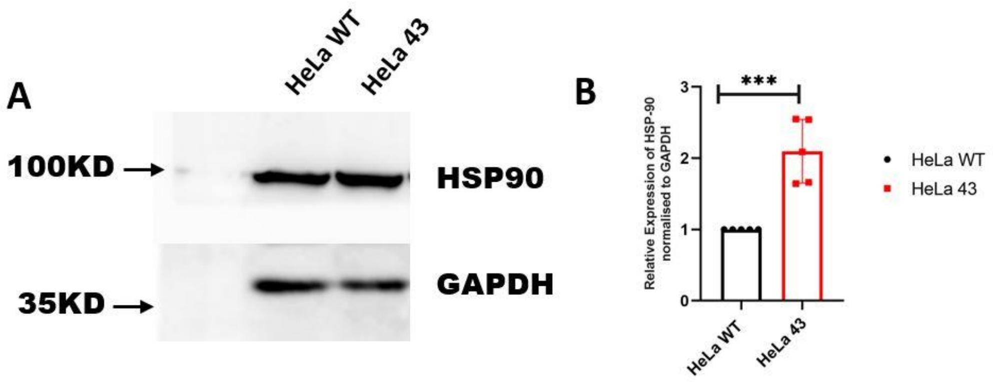

Fig. S5. Higher Abundance of HSP-90 in Hela 43 cells.

Western blot analysis of Hela WT and Hela 43 cell lysate (A) indicate higher abundance of HSP90 protein. Graph (B) depicts relative expression of HSP 90 in HeLa WT and Hela 43. Asterix (*) represents differences that are statistically significant by Student's unpaired t-test analysis (***P<0.001). The error bars represent SEM.

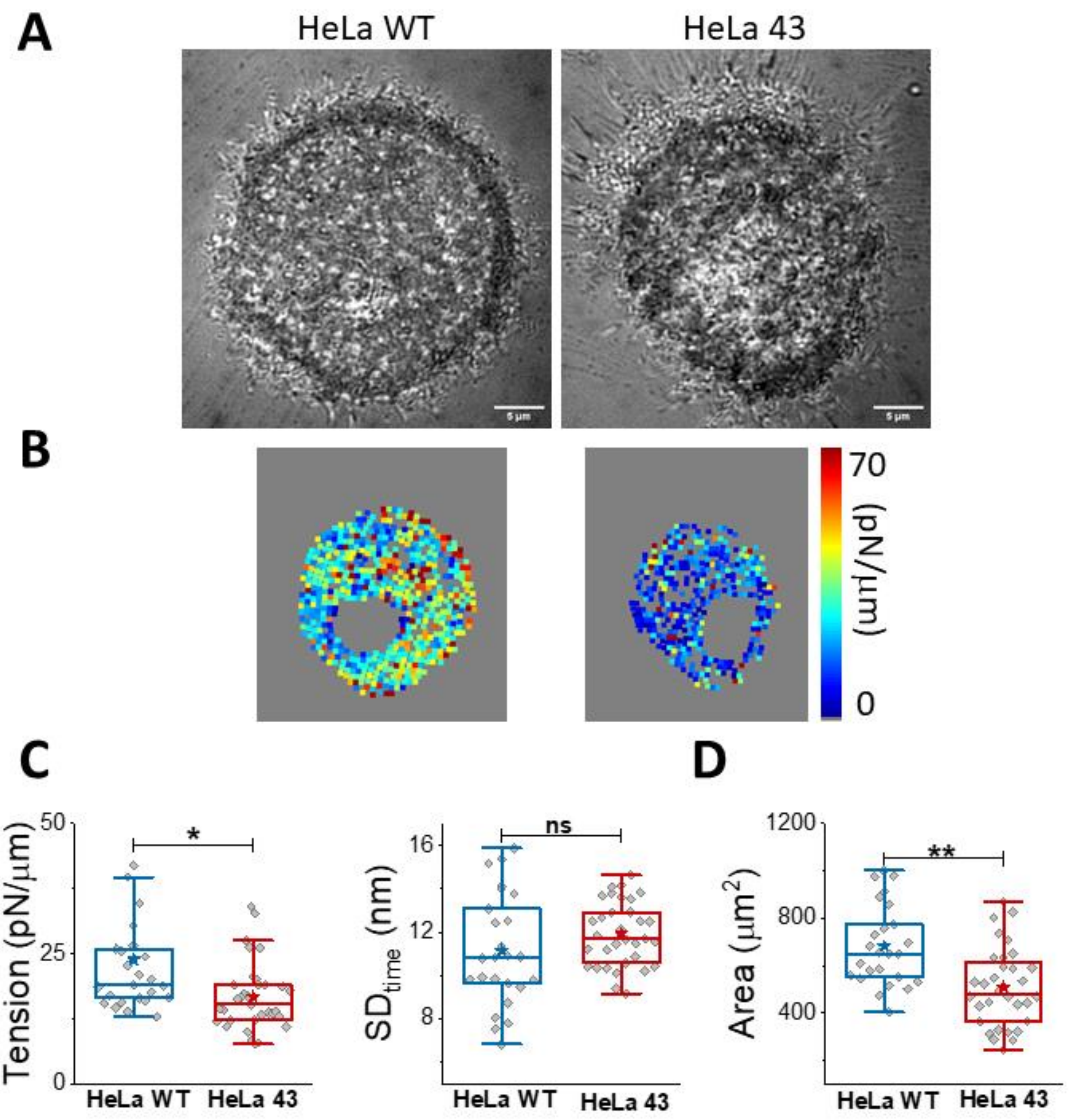

Fig. S6. Comparison of tension between HeLa WT and HeLa 43 with same morphology. (A) Representative IRM images of micropatterned in Cir 15 with the diameter of 30 μm of HeLa WT and HeLa 43. (B) Tension map of Represented IRM imaged cells in A. (C) Quantification of fluctuation-tension and fluctuation amplitude in HeLa WT and HeLa 43 cells; Number of cells: HeLa WT: 25, HeLa 43: 33. (D) Spread area plot for HeLa WT and HeLa 43 micropatterned cells.For statistical significance, the Mann-Whitney U test was performed, * denotes p values < 0.05, and ** denotes p value < 0.01. Representative of two independent experiments. Scale bar = 5 μm.

Biology Open • Supplementary information

Table S1. HeLa WT

ρ*	=					
	**Angle**	**Distance**	**Area**	**Circ.**	**Perim.**	**Sh.Ind.**
**Angle**	1	-0.3718	0.0648	-0.083	0.1008	0.1162
**Distance**	-0.3296	1	0.3276	0.3163	-0.3679	-0.3235
**Area**	0.0648	-0.3276	1	-0.5093	0.9218	0.4906
**Circ.**	-0.083	0.3163	-0.5093	1	-0.7882	-0.9877
**Perim.**	0.1008	-0.3679	0.9218	-0.7882	1	0.7842
**Sh.Ind.**	0.1162	-0.3235	0.4906	-0.9877	0.7842	1
**p****	=					
	**Angle**	**Distance**	**Area**	**Circ.**	**Perim.**	**Sh.Ind.**
Angle	1	0.0000	0.4693	0.3537	0.2596	0.1934
**Distance**	0.0002	1	0.0002	0.0003	0.000	0.0002
**Area**	0.4693	0.0002	1	0	0	0
**Circ.**	0.3537	0.0003	0	1	0	0
**Perim.**	0.2596	0.000	0	0	1	0
**Sh.Ind.**	0.1934	0.0002	0	0	0	1

* : denotes correlation coefficient
**: denotes the p-value

Table S2. HeLa 43

ρ*	=					
	**Angle**	**Distance**	**Area**	**Circ.**	**Perim.**	**Sh.Ind.**
Angle	1	-0.002	0.1568	-0.0369	0.1258	0.0291
**Distance**	-0.002	1	-0.1249	0.3347	-0.2120	-0.3146
**Area**	0.1568	-0.1249	1	-0.3282	0.9168	0.3009
**Circ.**	-0.0369	0.3347	-0.3282	1	-0.6619	-0.9903
**Perim.**	0.1258	-0.2120	0.9168	-0.6619	1	0.6443
**Sh.Ind.**	0.0291	-0.3146	0.3009	-0.9903	0.6443	1
**p***	=					
	**Angle**	**Distance**	**Area**	**Circ.**	**Perim.**	**Sh.Ind.**
Angle	1	0.9854	0.147	0.7342	0.2458	0.7888
**Distance**	0.9854	1	0.2491	0.0015	0.0487	0.0030
**Area**	0.147	0.2491	1	0.0019	0	0.0046
**Circ.**	0.7342	0.0015	0.0019	1	0	0
**Perim.**	0.2458	0.0487	0	0	1	0
**Sh.Ind.**	0.7888	0.0030	0.0046	0	0	1

* : denotes correlation coefficient
**: denotes the p-value

Table S3. Statistics of velocity as plotted in Fig. 5. All values are expressed in µm/min

Cell Type	Parameters	n	Mean	SD	SEM	Median
**HeLa WT**	Between 0-4 hr_control	8	0.0179	0.0047	0.0017	0.0184
	Between 0-4 hr_BFA	8	0.0144	0.0034	0.0012	0.0131
**HeLa 43**	Between 0-4 hr_control	8	0.0111	0.0051	0.0018	0.0102
	Between 0-4 hr_BFA	8	0.0098	0.0044	0.0015	0.0103
**HeLa WT**	Between 4-8 hr_control	8	0.0236	0.0061	0.0022	0.0242
	Between 4-8 hr_BFA	8	0.0134	0.0032	0.0011	0.0137
**HeLa 43**	Between 4-8 hr_control	8	0.0126	0.0036	0.0013	0.0113
	Between 4-8 hr_BFA	8	0.0065	0.0042	0.0015	0.0063
**HeLa WT**	Between 8-12 hr_control	8	0.0357	0.0106	0.0038	0.0336
	Between 8-12 hr_BFA	8	0.0166	0.0038	0.0014	0.0150
**HeLa 43**	Between 8-12 hr_control	8	0.0174	0.0031	0.0011	0.0163
	Between 8-12 hr_BFA	8	0.0012	0.0045	0.0016	0.0022

Biology Open • Supplementary information
